# Investigating Cell Signaling with Gene Expression Datasets

**DOI:** 10.24918/cs.2019.1

**Published:** 2019-01-03

**Authors:** James Wachira, Cleo Hughes-Darden, Asamoah Nkwanta

**Affiliations:** 1 Department of Biology, Morgan State University, 1700 E. Cold Spring Lane, Baltimore, MD 21251.; 2 Department of Mathematics, Morgan State University, 1700 E. Cold Spring Lane, Baltimore, MD 21251.

## Abstract

Modern molecular biology is a data- and computationally-intensive field with few instructional resources for introducing undergraduate students to the requisite skills and techniques for analyzing large data sets. This Lesson helps students: (i) build an understanding of the role of signal transduction in the control of gene expression; (ii) improve written scientific communication skills through engagement in literature searches, data analysis, and writing reports; and (iii) develop an awareness of the procedures and protocols for analyzing and making inferences from high-content quantitative molecular biology data. The Lesson is most suited to upper level biology courses because it requires foundational knowledge on cellular organization, protein structure and function, and the tenets of information flow from DNA to proteins. The first step lays the foundation for understanding cell signaling, which can be accomplished through assigned readings and presentations. In subsequent active learning sessions, data analysis is integrated with exercises that provide insight into the structure of scientific papers. The Lesson emphasizes the role of quantitative methods in research and helps students gain experience with functional genomics databases and data analysis, which are important skills for molecular biologists. Assessment is conducted through mini-reports designed to gauge students’ perceptions of the purpose of each step, their awareness of the possible limitations of the methods utilized, and the ability to identify opportunities for further investigation. Summative assessment is conducted through a final report. The modules are suitable for complementing wet-laboratory experiments and can be adapted for different courses that use molecular biology data.

## INTRODUCTION

Cell signaling is a fundamental property of living systems, and it is characterized by complexity arising from both direct and indirect interactions of signaling molecules. In multicelular organisms, homeostasis maintains optimal internal conditions for the survival of the organism, such as metabolic states and blood pressure, through both physiological and behavioral adaptations. At the molecular level, physiological responses are regulated through chemical messages released by different cells, including neuronal and endocrine cells. Thus, understanding the mechanisms of action of chemical messages, which is a part of cell signaling, is critically important to different subfields of biology. Consequently, cell signaling is covered in both lower- and upper-division courses in undergraduate biology curricula. Foundational knowledge is available in excellent publicly available resources, such as Scitable (https://www.nature.com/scitable) and iBiology (https://www.ibiology.org/biology-videos/).

Cell signaling is a key topic in both introductory and upper division biology textbooks. However, signal transduction pathways are commonly presented as linear, hierarchical events with just a handful of proteins. This approach likely arises for historical reasons: the canonical signal transduction pathways were discovered with non-throughput techniques mostly involving the study of individual genes and their products. However, the recent availability of high throughput molecular datasets presents an opportunity for evaluating additional interactions between different proteins and pathways. Indeed, recent studies have suggested the involvement of secondary signaling pathways as well as cross-talk between different receptors (1–3). This additional complexity remains a largely unexplored area of cell biology with unknown physiological implications (4). In this Lesson, the students first learn the basic organization of the signaling pathways to lay a foundation for a more in-depth systems view of the integrated nature of the molecular functioning of cells. Further, students gain skills for data analysis, representation, and visualization through engaging in labs based on the Rat Genome Database (RGD) (https://rgd.mcw.edu/) and the National Center for Biotechnology Information (NCBI) (https://www.ncbi.nlm.nih.gov/) (5,6).

Efficacy of the traditional “lecture method” of instruction has been challenged in recent years by studies indicating that students achieve significantly higher gains in learning if engaged in active learning activities (7–9). The principles of this approach to science education are embodied in the Scientific Teaching framework (10) that emphasizes: (i) the need to engage students in scientific inquiry in classroom activities; (ii) the use of cognitive science principles in the design of instruction; (iii) the importance of diversity and inclusive teaching practices in learning environments; and (iv) the application of assessments and resulting data to improve instruction (7). To accomplish these principles, instructional materials that reflect the authentic conduct of scientific studies are needed for different content areas and at different stages of the curriculum. The activities should be designed to transmit foundational knowledge while engaging students in meaningful experimentation or data interpretation.

The availability of publicly available molecular biology data has created opportunities for developing instructional materials that can encompass key concepts of cell biology, the scientific process, and data analysis. For example, molecular visualization of 3D protein structures coupled with sequence alignments and molecular docking were used to teach the concepts of protein structure and function (11). In the process of learning the principles of protein structure, students acquired computational molecular biology skills. A bioinformatics module in which students used the databases to generate a new understanding of protein function was demonstrated to be effective in promoting engagement and acquisition of research skills by undergraduate students (12). Baumler et al. reported on the effectiveness of a module that introduced students to the National Center for Biotechnology Information’s (NCBI’s) Basic Local Alignment Search Tool (BLAST) and multiple alignment of genomes in the context of analyzing the conservation of virulence factors in bacterial intestinal pathogens (13). The approach enhanced self-efficacy and the understanding of content knowledge. Educational benefits of using professional visualization software to explore and analyze “-omics” data in an undergraduate course have also been reported (14). In a semester-long computational genetics course using publicly available genomics data, students acquired competencies in bioinformatics tools and peer-peer collaboration that allowed them to co-author papers with their instructors (15). Other authors have developed learning activities that utilize genomics data to introduce undergraduate students to RNAseq sequence assembly and data analysis pipeline in the R statistical environment (16). In all, these and similar articles are geared towards transmitting both course knowledge and scientific research skills.

In this Lesson, we introduce students in the junior and senior years of study to RGD and NCBI’s resources through computer labs and assignments. The NCBI databases of interest at this level are PubMed (https://www.ncbi.nlm.nih.gov/pubmed/) for literature searches, the sequence repositories Gene (https://www.ncbi.nlm.nih.gov/gene/), Protein (https://www.ncbi.nlm.nih.gov/protein/), and Nucleotide (https://www.ncbi.nlm.nih.gov/nuccore/), and Gene Expression Omnibus (GEO) (https://www.ncbi.nlm.nih.gov/sites/GDSbrowser?acc=GDS4756). The RGD is used to retrieve curated signaling pathways to investigate the function of specific genes in the context of signal transduction pathways. At this stage, the students identify both the molecular activities associated with signaling proteins and their roles in signaling pathways. The gene entries in the RGD database link-out to NCBI databases and in the next stage students further explore the functions of specific genes by reading summaries provided by the databases, such as the Gene database, as well as published articles through PubMed. Through this activity, students learn to navigate public genomics resources and acquire scientific writing and presentation skills. Fundamentally, this stage resembles the scientific process of literature searches during the development of scientific projects and hypotheses. The Lesson is designed around a cancer molecular therapy and in the next stage, students learn the connection between signaling kinases and human diseases through reading curated articles in the On Mendelian Inheritance In Man (OMIM) database (https://www.ncbi.nlm.nih.gov/omim). In the final stage, they analyze, synthesize, and present primary data obtained from GEO Datasets. Note that there are many opportunities for incorporating wet laboratory experiments in addition to the computational activities depending on the availability of resources and time allocation. For example, from their data analysis, students may generate hypotheses about the effects of specific inhibitors on gene expression; they could then design primers and conduct real-time PCR experiments to test predictions of their hypotheses.

### Rationale

Signal transduction pathways coordinate diverse cellular processes such as growth, proliferation, metabolism, adherence, migration, and gene expression (17–19). However, the diversity of the concepts and the simplified presentations in many textbooks may leave students without an appreciation of the complexity of cell signaling, its consequences on gene expression, and the opportunities for further research and discovery (20). Furthermore, research on how students integrate molecular biology knowledge to cellular processes indicate that while students demonstrate knowledge of processes such as DNA replication, transcription, and translation, they often fail to link molecular processes to roles in cellular function (21). These authors proposed the integration of molecular mechanisms in the context of cellular functions in molecular and cellular biology courses (21). Indeed, traditional lectures are unlikely to fully convey the various levels of abstraction of signal transduction data and concepts in textbooks and other published literature. Thus, many students may complete an undergraduate program with limited understanding of cell signaling and lacking the skills needed to analyze primary data or to further explore the topic.

Cell signaling research has ushered in a new era of targeted cancer therapies, many of which disrupt growth factor receptors and downstream signal transduction molecules, such as protein kinases (22). The successfully targeted signaling kinases include EGFR, BRAF, MEK, BCR-Abl kinase, and, potentially, AKT/PKB (Table 1) (22–26). Because of the high prevalence of cancer, many students are familiar with its societal ramifications and recognize the need for better approaches to prevention and treatment of the disease. The extensive literature and publicly available molecular data on cancer (27–29) are readily available resources for teaching basic cell biology and research methodology using a topic that captures the interest of students.

Inquiry-based learning affords students the benefit of exploring different aspects of a problem in an integrated manner and creates a medium for teaching the process of science (30). Also, new advances in biology require increasingly higher levels of competence in data analysis skills. The need to better train undergraduate students in computational and quantitative methods in an integrated way is widely recognized (31,32). Biology undergraduate curricula typically include several mathematics courses; however, it is noted in the literature that in many cases mathematical skills are not easily applied to biological problems (33). Genomics datasets provide students with the opportunity to apply computational and statistical methods directly in the discipline using authentic research data. The feasibility of this approach is supported by the work of other authors who have relied on undergraduate researchers to curate genomics databases in diverse areas including neuropathology, Drosophila genetics, and plant science (34–36).

This activity is designed around the utilization of publicly available databases and gene expression analytical tools, such as GEO Datasets (29). Meta-analysis of data stored in GEO Datasets has led to additional insights into disease mechanisms (37,38), indicating that this approach has utility beyond student instruction. The purpose of this Lesson is to use these resources to explore cell signaling in a series of undergraduate computer laboratory sessions. We expect that the inquiry-based approach will help students consolidate their understanding of molecular biology and improve their scientific and quantitative skills.

### Intended Audience

This Lesson is designed for an upper level cell and molecular biology course. The preferred instructional approach is to provide a concise but comprehensive overview of cell biology during the first three weeks of the semester prior to this Lesson. During this period, the students are assigned readings that cover the first and fourth chapters of the textbook to introduce them to techniques in cell biology and cell structure and function (39). The readings are supplemented with links to short videos and articles that provide more detail and opportunities for inquiry-based learning (Supporting File S1: Foundational Knowledge). This foundation is helpful to make the labs in the Lesson comprehensible and to prepare students for inquiry-based learning.

Beginning in the junior year and certainly in the senior year of study, students enroll in specialized courses, such as immunology, that not only apply concepts learned in survey introductory biology courses but also integrate diverse concepts ranging from molecular mechanisms to cellular processes. For example, students may have learned cellular signaling in the context of glycogen mobilization and transcription as a process that leads to the expression of genes. In immunology and other upper-level courses, the same signaling modules may be introduced in a different context and it becomes important for the learner to appreciate the modularity of signaling components and the different interconnections of signaling and effector proteins. However, research suggests that integration of molecular and cellular biology knowledge from the molecular mechanistic level to the higher level cellular processes poses difficulties for students (21). This Lesson, therefore, provides an opportunity for integrating different molecular and cellular biology concepts that can be applied to different subfields of biology.

By design, the Lesson’s components described here encourage students to review topics covered in lower level biology courses and to read advanced material from selected curated databases such as RGD (5). Thus, it is compatible with courses that rely on knowledge gained in introductory biology courses and that seek to deepen the knowledge of molecular processes. The information in these databases engage students in the deep reading of complex passages and integrate topics as diverse as transcription and physiology.

### Required Learning Time

This Lesson contains two modules, each of which requires two 3-hour lab sessions (Table 2). Note that the videos (iBiology) (https://www.ibiology.org/ibioseminars/cell-biology/robert-lefkowitz-part… and https://www.ibiology.org/biochemistry/protein-kinase/) and GEO tutorial (NCBI) (https://www.ncbi.nlm.nih.gov/geo/info/datasets.html) could be assigned to students in advance as discussed below. In the first module, 2–3 hours of instruction time are spent on establishing foundational knowledge in cell signaling, accomplished through assigned readings, video presentations, and classroom discussions. In addition to a textbook, instructors can draw on many freely accessible reference materials for teaching such as book chapters available through NCBI (http://www.ncbi.nlm.nih.gov/books/NBK21517/) (40), Scitable (https://www.nature.com/scitable/topic/cell-communication-14122659), and particularly important for this Lesson are the two selected iBiology videos (https://www.ibiology.org/biochemistry/protein-kinase/ and https://www.ibiology.org/cell-biology/g-protein-coupled-receptors/). To scaffold the learning and class discussions, a list of questions is provided to students in advance, which gives them the opportunity to prepare to respond to questions in class (see Supporting File S5: Example of a Worksheet for Guiding Group Discussion). The worksheet questions also provide a basis for structuring group discussions and it serves as an outline for self-study. The next three hours in this module are spent on a computer lab (see Supporting File S3: Cell Signaling Activity).

In the second module, students are instructed on the use of GEO with a demonstration. This step should take about 30 minutes. In the next step, students retrieve and analyze data (1 hour). One important point to note is that the data can be analyzed either with GEO DataSets analysis tools or through GEO2R (https://www.ncbi.nlm.nih.gov/geo/geo2r/). The next hour is spent introducing students to data analysis with geWorkbench and tutorials on the usage of the software package are available through the program developers’ website (http://wiki.c2b2.columbia.edu/workbench/index.php/Home) (41). After the introduction, the analysis can be completed in class as groupwork or as an assignment (Supporting File S4: Functional Genomics Activity). The last step is the integration step whereby the identified genes are mapped to specific pathways using DAVID, the Database for Annotation, Visualization and Integrated Discovery (https://david.ncifcrf.gov/home.jsp) and takes 2 hours (42). Eventually the students will submit a lab report covering the two modules and the last 45 minutes should be spent on discussing the structure of a scientific paper.

### Pre-requisite Student Knowledge

The Lesson is designed for juniors/seniors majoring in biology. Students should have a basic understanding of cell signaling, which could also be introduced or reviewed via lectures (Supporting File S2: Cell Signaling Lecture Outline). This entails having background knowledge of the stages of a canonical G protein-coupled receptor, such as an adrenergic receptor, signaling pathway. Many free resources are available for introducing this topic (https://www.nature.com/scitable/topic/cell-communication-14122659 and https://cnx.org/contents/GFy_h8cu@9.85:H4oMpCSi@7/Signaling-Molecules-and-Cellul). This information is well documented in introductory biology textbooks for biology majors, for example Campbell Biology (43). The different stages of signaling are reception, transduction, and response, which can be illustrated with examples from topics that are already familiar to students, such as energy metabolism to build on preexisting knowledge. With this background, comparison could then be made with an example of another class of receptors, such as receptor tyrosine kinases (Supporting File S2: Cell Signaling Lecture Outline) and there many excellent free resources, for example (https://www.khanacademy.org/science/biology/cell-signaling/mechanisms-of…).

To progress and benefit from this Lesson, students should also be familiar with biological sequences (DNA, RNA, protein), as well as have fundamental computational skills, such as data manipulation with spreadsheets. In particular, students may be unfamiliar with the format of representing mRNAs with T’s instead of U’s in GenBank and the fact that, in many cases, higher eukaryotes’ genes are represented by many mRNA isoforms in the database. Thus, the concepts of alternative splicing, coding and non-coding strands of DNA, and the relationships between the structures of genes and the proteins they code for should be reviewed prior to undertaking the more advanced parts of this Lesson. These topics are covered in introductory biology textbooks and there are other freely available materials on the Web (https://www.ncbi.nlm.nih.gov/books/NBK21132/, https://www.khanacademy.org/science/biology/gene-expression-central-dogm…, and https://www.ibiology.org/speakers/melissa-moore/).

### Pre-requisite Teacher Knowledge

The Lesson involves retrieving, manipulating, and analyzing biological sequences and gene expression data. The purpose of the first module is to develop a general understanding of cell signaling through the study of specific curated pathways. The learning activities are based on the Pathways dataset of the RGD (5). Whereas navigation through the pages is straightforward, it is important for the instructor to become familiar with the technical terminology of the field. A helpful video on navigating the diagrams of the pathways and the associated terminology is presented at the RGD website (https://rgd.mcw.edu/wg/home/rgd_rat_community_videos/molecular-pathway-d…). Users of the interactive pathways obtain further information by linking out to other databases and tools, such as the NCBI’s Gene database and Map Viewer. It is necessary for instructors to be conversant with such external sites before guiding students through this module. The first lab provides an active learning protocol to enable instructors to cover topics in cell signaling and the organization of eukaryotic genes and proteins (Supporting File S3: Cell Signaling Activity). In the final step, the students are asked to reflect on a pathway and develop hypotheses regarding particular genes and diseases. Knowledge of how mutations affect protein structure and function is essential. This last step of module 1 lays the foundation for inculcating the principles of hypotheses testing using publicly available genomics data as explained in the next module and the instructor should have a general awareness of the implications of multiple hypothesis testing when dealing with high-dimensionality data, such as genomics data (https://discover.nci.nih.gov/microarrayAnalysis/Microarray.Home.jsp) (47–49). This module can be used to launch a wet laboratory activity which tests the effects of pharmacological agents on cellular processes; in this case, the instructor would need to be familiar with cell culture techniques (44–46).

The instructor should be familiar with basic bioinformatics techniques for retrieval and analysis of biological sequences. As indicated above, sequences for signaling proteins in the RGD can be retrieved for further analysis through GenBank. Since this is an upper division course Lesson, it is imperative that students begin to learn cell and molecular biology concepts in an integrated manner as opposed to individual topics. To accomplish this in an interactive way, they are guided through the different sequence databases. The NCBI databases are extensive and well documented (50). Videos and tutorials are available through the NCBI website (https://www.ncbi.nlm.nih.gov/home/learn/). For example, information flow from DNA to protein and the complexities arising from alternative splicing and differential use of initiation codons can be rediscovered by navigating between the Genome Data Viewer (https://www.ncbi.nlm.nih.gov/genome/gdv/) and the RefSeq databases of both nucleotides and proteins (51). Having examined the relationships between a gene and the encoded mRNAs and proteins, the next level of interest could be the domain organization of a protein to gain an insight into function. This can be accomplished through the Conserved Domains Database (CDD) (52). In the planning phase, the instructor will identify a path through the databases that highlights specific concepts of interest and then use the NCBI’s training resources to become acquainted with the relevant procedures.

To lead into the second module, the instructor should also be familiar with functional genomics experiments and data as well as software packages for analyzing functional genomics data. The first step is to gain an overview of the GEO database through NCBI’s online videos, training materials, and publications (https://www.ncbi.nlm.nih.gov/home/learn/) (53). The goal of a microarray experiment is to reveal the transcripts profile of an experimental system at a global level and over the years approaches for the analysis of the resulting data have been developed. Slonin and Yanai (47) have provided a guide for microarray data analysis that suffices for this Lesson. This Lesson uses geWorkbench for data analysis and developers of the program also provide full documentation and tutorials through a website (http://wiki.c2b2.columbia.edu/workbench/index.php/Home). Depending on the dataset chosen for the analysis, an annotation file may need to be downloaded from a microarray platform manufacturer’s website, for example Affymetrix (http://www.affymetrix.com/products/index.affx) and the links for the annotation files are indicated in the respective GEO records.

## SCIENTIFIC TEACHING THEMES

### Active learning

The Lesson was developed for a class of 30 students working in groups of three to five. The students organize themselves into groups; however, the membership of individual groups does change from time to time. Although students work in groups, each student submits their own individual work for grading (see Supporting File S3: Cell Signaling Activity and Supporting File S4: Functional Genomics Activity). The steps in both documents require active participation in reading passages, retrieving information and data, and analyzing and presenting data.

**Figure 1.**
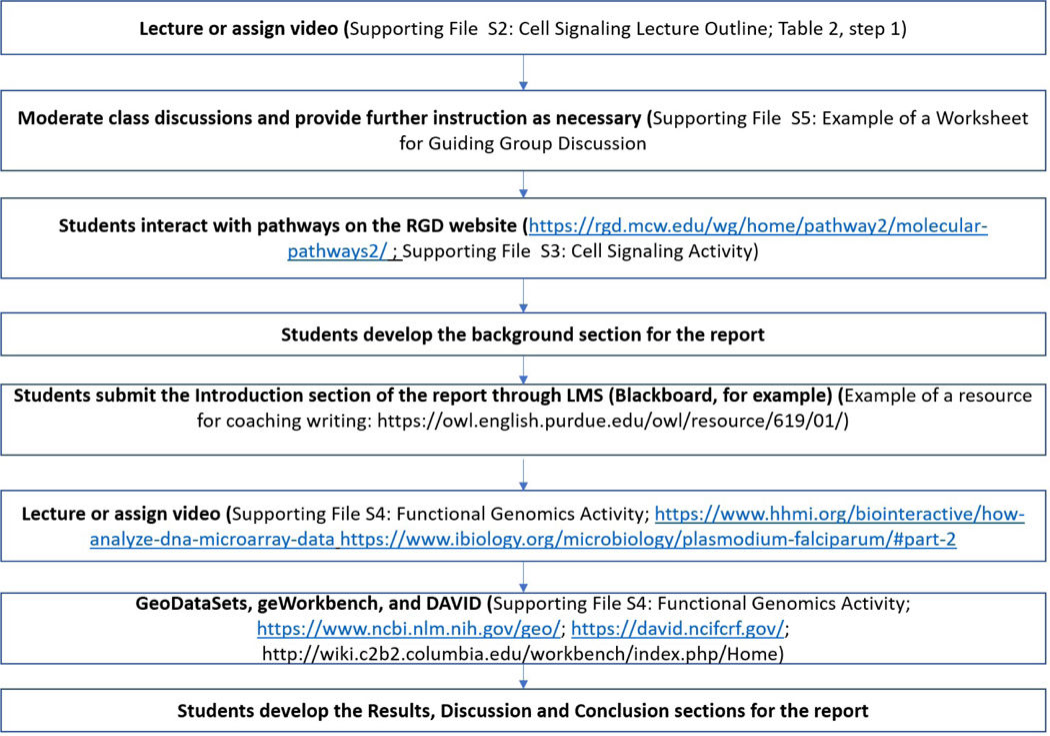
Steps in the two modules encompassing 4 lab sessions. The detailed steps are presented in Table 2.

The steps for engaging students in active learning activities are presented in Figure 1 and Table 2.

The instructor introduces the content and tools to enable students to effectively read the literature and analyze data in the initial steps of the Lesson. However, it may be necessary to reiterate some concepts as students discover new information to reinforce the learning and enhance self-efficacy. The second step is accomplished through reading and interacting with pathways data on the RGD website (5). At this stage, students identify each of the steps on the signal transduction pathway by clicking on specific proteins to retrieve curated functional information. For example, clicking on a receptor may yield further information on classification and both natural and pharmacological ligands. This could lead to a discussion on the different functional outcomes of ligand-receptor interaction and the concepts of agonism and antagonism, including medical applications. A similar activity on a signaling molecule could yield information on biochemical activity, such as kinase or adenylyl cyclase activity. This then leads to a discussion on signal transduction and amplification. The instructor visits the groups during groupwork to encourage discussion and to offer assistance as necessary. The small-group discussions are interrupted periodically to allow for report out sessions in which different groups take leadership in explaining a biological concept to the class. The open-ended nature of this Lesson allows the instructor to tailor the depth and breadth of coverage to the existing background knowledge of his or her students and the desired level of difficulty. This module then serves to develop the Background section of a report and it creates many opportunities for students to discuss the concepts within groups and to report the groups’ synthesis to the class. Given that each student will be graded on submitted work, students are usually very motivated to cooperate in finding as much information as possible. Group presentations are awarded participation points. At the conclusion of this section, and consistent with contemporary approaches to research, the students develop a hypothesis on the outcomes of inhibition of a pathway on a biological process. The end points are chosen based on feasibility of implementation.

In the second module of the Lesson (see Supporting File S4: Functional Genomics Activity), microarray data from NCBI’s GEO DataSets (53) is analyzed following two approaches. In the first approach, students use data analysis tools provided by GEO. This includes hypothesis testing with t test and clustering. At this point, the different levels of stringency afforded by p-values are demonstrated and discussed. Experimental design is also examined with the students being asked to choose different samples to serve as either case or control. In the second approach, the students download the datasets for analysis with geWorkbench (41). In both modules, the students enter their responses in the spaces provided in Supporting File S4.

**Figure 2.**
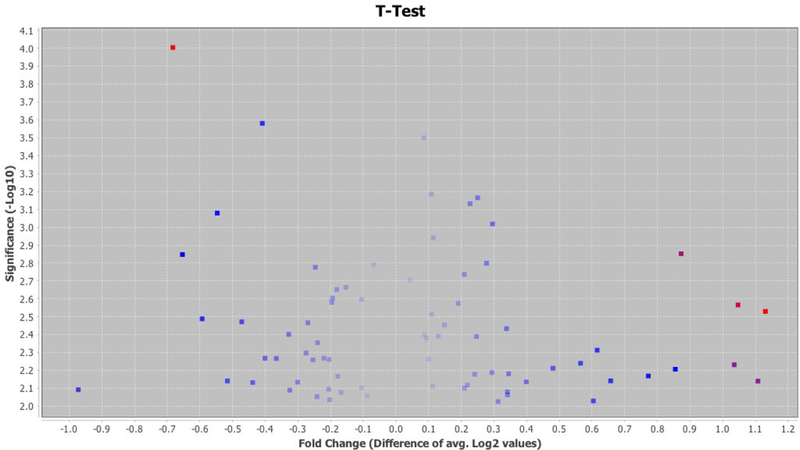
Volcano plot of gene expression analysis results. p-values are plotted on the Y-axis and the fold changes in gene expression levels on the X-axis. Volcano plots are useful for simultaneously visualizing both the significance levels and fold changes in gene expression data. Spots that fall in the top right and top left quadrants identify genes with the lowest p-values and highest fold changes in expression levels.

**Figure 3.**
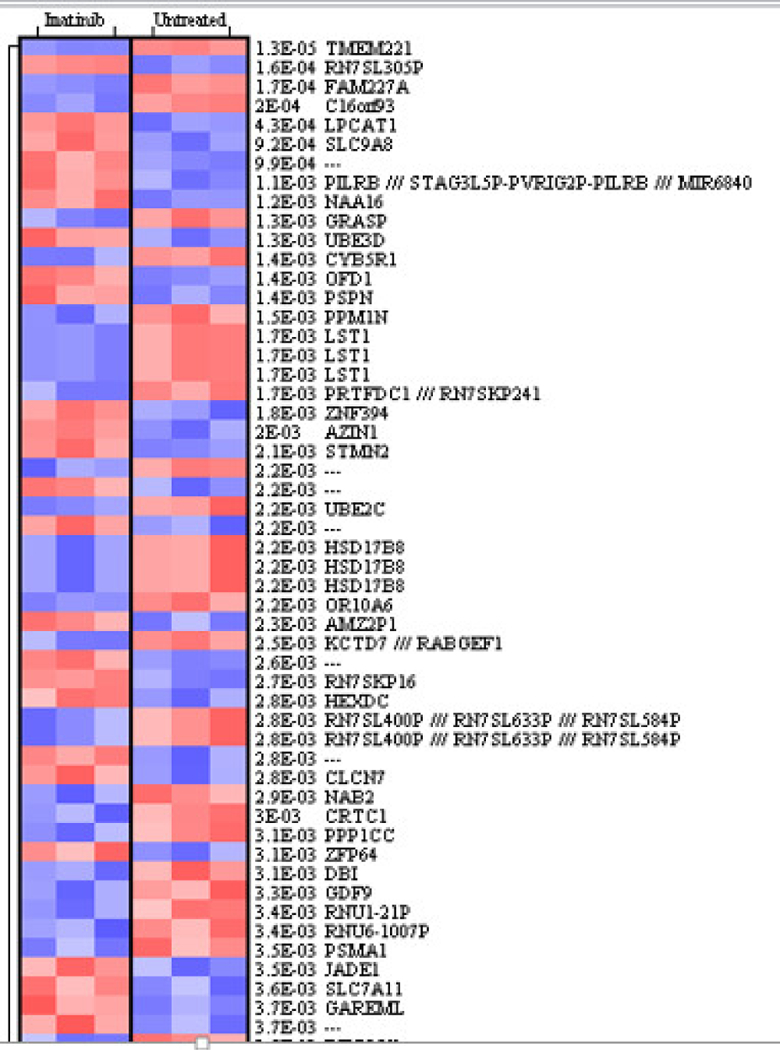
Heatmap showing differentially expressed genes. Heatmaps are useful in visualizing high-throughput gene expression data because they provide a visual representation of patterns in the data.

Through these activities, the students work in groups to develop the key points for the lab report while receiving feedback at different steps (Figure 1). The report then includes results of data analysis using software as well as figures summarizing the data (see Figures 2 and 3).

### Assessment

The Lesson is designed to use publicly available molecular biology resources to teach both the process of science and a key topic in cell biology. At the beginning of the modules, students answer specific questions related to NCBI resources to gauge their prior knowledge of the application of computational methods in molecular biology (Supporting File S6: Laboratory Skills Survey). As indicated above, there are many molecular biology databases and software packages on the Web including the resources provided by the NCBI, RGD, Mouse Genome Informatics (MGI) (http://www.informatics.jax.org/), European Molecular Biology Laboratory-European Bioinformatics Institute (EMBL-EBI) (https://www.ebi.ac.uk/), among others, (5,6). Our assessment helps to collect information on the baseline knowledge of molecular biology techniques and complementary bioinformatics tools, structure instructor led demonstrations, and to provide students with an overview of the learning objectives of the Lesson. In the initial phases, writing coherent passages from scientific literature are developed. When students review the RGD pathways, they report on the functions of the proteins involved by reading from other resources (see Supporting File S3: Cell Signaling Activity, question 1). The instructions for students list the elements of an Introduction section of a scientific paper. An explanation of how to coach students on scientific writing is given under Notes for instructors (see Supporting File S3: Cell Signaling Activity, at the end of the introduction section) and the instructor can use other resources, for example the Elsevier guide (https://www.elsevier.com/connect/11-steps-to-structuring-a-science-paper…). Short answer questions are interspersed within the procedures (see Supporting File S3: Cell Signaling Activity and Supporting File S4: Functional Genomics Activity). These questions seek to integrate the analytical stages of the Lesson with the biological facts or knowledge and to develop a habit of thinking in an integrated manner. For example, after reviewing the PI3K-AKT pathway on RGD the students answer questions on the functions of AKT domains (see Supporting File S3: Cell Signaling Activity, question 4). One of the follow up questions allows students to discuss the mechanisms of activation of AGC kinases to which AKT belongs (see Supporting File S3: Cell Signaling Activity, question 5). Implicit in this question is the evolutionary relatedness of these kinases, providing an opportunity to discuss the concept of evolution from a molecular perspective. The second module is devoted to data analysis and the questions relate to hypothesis testing (Supporting File S4: Functional Genomics Activity). For example, students formulate a hypothesis prior to data analysis, select groups of samples to compare, and select a statistical test to use and at each step they provide appropriate justifications by responding to questions (see questions 2, 5, 6, and 7). Each question in the protocols is graded by the instructor and 20% of the grade for each Module comes from participation through class discussions. In addition to written answers, students generate figures and interpret gene expression data. At the end of the modules, they submit a final report that aggregates the different stages of the Lesson with corrections as suggested by the instructor.

### Inclusive teaching

The Lesson engages students in experimentation with software, thinking activities that require the integration of disparate pieces of information, experimentation, and reflection. In their totality, these activities engage students with differing learning preferences. In our experience, students are proficient in finding information on the Web; however, in many cases they require training in identifying the most salient points, and in writing and paraphrasing scientific content. The process of gathering information and re-writing is fundamental to internalization and care should be taken in the selection of materials to ensure the appropriate level of difficulty since students may not be able to meaningfully write if the material is too complex. Depending on the pathway selected, examples should be drawn from students’ lived experiences. This promotes connection with the subject matter. Assigned readings from the relevant sections of the textbook provide the baseline knowledge for further exploration. Another excellent source for foundational knowledge in cell biology in general and cell signaling in particular is Scitable (https://www.nature.com/scitable).

Through manipulation of the pathways and in a group setting, students explore important scientific concepts that touch on gene structure and function. The opportunity to cooperate with peers and to meaningfully contribute to the groups accomplishments builds confidence and work ethic. Given the availability of audiovisual media resources in this area, such as videos and educational articles from authoritative websites (for example, https://www.ibiology.org/biology-videos/, https://www.nature.com/scitable, and https://www.hhmi.org/biointeractive/about-biointeractive), students can be engaged through different methods that make learning interactive and appealing to students with different learning styles. Also, these materials allow students who start at different levels of understanding the opportunity to attain the desired level of mastery if they are willing to put in the effort. The links for learning materials of different levels of complexity are posted on the Learning Management System (LMS), which allows students the opportunity select different starting points based on background knowledge. The instructor describes these materials to give the students a general understanding of the different levels of difficulty. Based on this, advanced students can progress to research articles quickly whereas other students would spend more time on the videos and introductory websites before progressing to research articles.

Overall, the Lesson engages students in hands-on activities, incorporates student led discussions, and draws on student- lived experiences. Through this approach students learn the required content and gain computational skills from the instructor and through cooperation with their peers.

## LESSON PLAN

### Introductory Instruction

An instructional period on cell signaling of two to three hours precedes the activities of this Lesson with the material being drawn essentially from the course textbook and iBiology videos. The course textbook for the class is Becker’s World of the Cell and the accompanying website, MasteringBiology (39). Testing is an important activity during learning (54) and quizzing programs can help enforce factual learning. Instructors can find adequate teaching resources through the NCBI and iBiology as indicated in the previous sections. During the lecture portion of the Lesson (Table 2 and Supporting File S2: Cell Signaling Lecture Outline), the instructor introduces the tenets of cell signaling, including the pertinent terminology, such as ligands, receptors, second messengers, post-translational modifications, signal transduction, signal amplification, and cellular responses. The basics are provided in chapter 23 of the above text and it is only necessary to cover one pathway, such as a paradigmatic G protein-coupled receptors pathway, as a general model of the hierarchy of signaling. This information is also available through lectures on iBiology (https://www.ibiology.org/biochemistry/protein-kinase/ and https://www.ibiology.org/cell-biology/g-protein-coupled-receptors/). The purpose of the lecture is to convey sufficient information on the organization of signaling pathways and their roles in the molecular functioning of cells to enable students to better learn the underlying mechanisms of cell signaling and cell biology research approaches interactively using public bioinformatics resources. The students are asked to read assigned textbook sections to consolidate their understanding of the fundamental concepts throughout the duration of the Lesson.

#### Module 1

In the first hands-on activity, students explore a key signal transduction pathway in the control of cell growth and proliferation and one that is commonly mutated in cancers. Though the instructor could choose from many different pathways, this Lesson focuses on the PI3K/AKT pathway (Supporting File S3: Cell Signaling Activity). The RGD or KEGG databases are very suitable for this exercise as the gene entries are linked to the NCBI’s Gene database (5,55). By following different links including Online Mendelian Inheritance In Man (OMIM) (https://www.ncbi.nlm.nih.gov/omim), students become familiar with not only the molecular and physiological functions of the gene products of interest, but also with gene-disease associations. They also gain an insight into authoritative sources of biomedical information and the role of informatics in biomedical research. These activities are accompanied by in-class exercises (Supporting File S5) in which the students answer very specific questions on cell signaling and engage in small group discussions. Depending on time constraints, the students can answer questions related to mutation/disease associations and research further the relevant scientific evidence.

#### Module 2

The students analyze functional genomics data using the online GEO analysis tools and then with the JAVA based geWorkbench software (41,53). The dataset chosen for this activity is derived from a microarray study on the effect of treatment of leukemia with imatinib, a BCR-Abl protein kinase inhibitor (56). Abelson kinase (Abl) kinase is a key regulator of growth and development and the oncogenic form, BCR-Abl kinase, is the key driver of chronic myeloid leukemia (57). At the molecular level, Abl functions downstream of cell surface receptors and upstream of Ras and phosphatidylinositol 3’-kinase (PI 3-kinase) (58,59). Other evidence suggests that it acts as scaffold protein in the assembly of signaling complexes and it also serves to link signal transduction pathways to the cytoskeletal responses (60,61).

After instructor guided group work on hypotheses testing and experimental design (Supporting File S4: Functional Genomics Activity), the students generate new hypotheses based on the abstract of the article accompanying the dataset and proceed to select samples for comparative analysis. Note the important activity of critically reading the abstract to gain insight into scientific writing style. The data are subjected to Student’s t test analysis at different p-values and the instructor may also suggest ANOVA. The results of the analyses are used to review and reinforce quantitative skills gained in other courses. For more advanced students, such as 400 level courses, other analytical methods, such principal component analysis, can be introduced. In the final stage of the Lesson, students visualize the results of the analysis and draw conclusions from the data. This Lesson is geared towards cell signaling through data analysis and either microarray or RNA-seq data could be used in the activities.

Examples of student- generated figures are shown in Figures 2 and 3. High-dimensional data requires special techniques for data mining and visualization and two widely used techniques for genomics data are the volcano plot and heatmaps. Both figures were generated from the microarray data with geWorkbench. The results of the t test were represented graphically with these techniques for visualization and identification of genes exhibiting significantly different expression between untreated and treated groups. In Figure 2, the level of significance was transformed to negative log base 10, which generates positive values with the highest levels of significance yielding larger numbers. The fold change was expressed as log base 2 and plotted on the X-axis. Thus, volcano plots place the genes with the highest fold changes and significance in the top left (downregulated genes) and top right (upregulated genes) quadrants. Heatmaps use a color scale to highlight differences and trends in the data.

## TEACHING DISCUSSION

The main findings of the New Biology Committee under the “Vision and Change in Undergraduate Education, A Call to Action” initiative centered on the transformative nature of newer technologies in scientific research, the increasing complexity of biomedical data, and the evolution of biological sciences to an interdisciplinary field (http://visionandchange.org/finalreport/). These trends must be taken into consideration in the training of the next generation of the biomedical workforce. The Lesson encapsulates these themes by engaging undergraduate students in analysis of data obtained with a high-throughput method. It provides instructors with the opportunity to discuss with the students how the choice of statistical methods and significance levels may influence the conclusions of a research study.

Different threshold-levels and normalization methods are available in geWorkbench. Students can explore the data using alternative methods to gain a better understanding of the role of quantitative methods in discovery. An understanding of the effects of different data handling methods on scientific conclusions can be demonstrated in the activities. The Lesson also introduces the importance of data visualization in research. In addition to deepening their knowledge of cell and molecular biology, students likely acquire critical computational skills for use in advanced studies.

In general, the students rated these computer lab activities as being both engaging and challenging. Importantly, they reported that the activities helped them better understand biological concepts. In a survey conducted at the beginning of the semester (Supporting Material S6), 58% of the students reported either not being familiar or having limited knowledge of the bioinformatics tools introduced in this Lesson. The number at the end of the semester was 34%. In the larger context of the labs section of the course, one student wrote how the skills gained made her feel prepared for an entry level position with a pharmaceutical company and another student remarked on how much the use of technology helped her learn cell biology.

Genomics techniques are at the cutting-edge of biomedical research and they generate very important data for discovery, clinical diagnosis, and biotechnology (62–66). This Lesson is amenable to adaptation to different biological problems that can be addressed with functional genomics. Thus, although our research interests are in the field of cell signaling from a biomedical perspective, which is reflected in the selection of the datasets, the protocol can be easily adapted to other fields, such as genomics and biotechnology. It is very applicable to developmental biology, immunology, and quantitative biology courses because publicly available datasets are available for these fields as well. Furthermore, the two modules can be used independently of one another to create shorter labs depending on class needs. On the other end of the spectrum, it is also possible to add a wet lab module or to use in-house generated data to create a more extended, Course-based Undergraduate Research project.

## Supplementary Material

S1

S2

S3

S4

S5

S6

## Figures and Tables

**Table 1.** Signaling kinases as targets for cancer treatment.

Molecular Target	Drug	Reference
Epidermal growth factor receptor (EGFR)	Gefitinib and erlotinib	(23)
Rapidly accelerated fibrosarcoma (RAF) family member BRAF	dabrafenib	(24)
Mitogen activated kinase MEK	Trametinib	(24)
Protein kinase B/AKT (AKT/PKB)	MK-2206, triciribine	(25)
Bcr-Abl kinase	imatinib	(26)

**Table 2.** Lesson activities and estimated time allocations.

Recommendations	Learning Outcomes	Assessment	Activity

**Module 1 (Two 3-hour Lab Periods, 6 hours total)**			
• Establish baseline knowledge of cell signaling using a lecture and/or assign a video from iBiology, for example, Protein Kinases: Structure, Function, and Regulation By Dr. Susan Taylor, UC San Diego; https://www.ibiology.org/speakers/susan-taylor/ or Seven Transmembrane Receptors, G protein coupled receptor kinases, and Beta-arrestins by Dr. Robert Lefkowitz, Duke University Medical Center; https://www.ibiology.org/?s=lefkowitz	The students will be able to: • Describe the stages and events of cell signaling. • Identify the role of cell signaling in cellular and organismal function.	Online quiz (multiple choice questions on cell signaling are selected from MasteringBiology database provided by the publisher)	• 2–3 hours: A combination of a short lecture of no more than 45 minutes and video presentations that are followed by class group discussions. It is important to scaffold the discussion with questions that cover the intended concepts. Note that the questions range from simple foundation knowledge questions to more complex questions that require knowledge integration and synthesis. • Assigned reading of textbook chapter. The reading need not cover the full chapter (chapter 23 in our text, Becker’s World of the Cell) and the students should read the introductory section and one pathway in detail. The suggested video is 1-hour long. However, it may be helpful to embed reflection questions as a separate hand-out (see Supporting File S2: Cell Signaling Lecture Outline and Supporting File S5: Example of a worksheet for guiding group discussion)
• Students work as lab groups and select a single pathway to study. At the end of the module each group makes a presentation to the class. • Groups of three students are ideal for this activity and the students should be encouraged to share the tasks.	The students will be able to: • Identify or describe the different stages of cell signaling. • Discuss the organization of different signal transduction pathways. • Explain how different enzymes achieve amplification of the signal during transduction. • Correlate signaling pathways to different cellular responses, such as metabolic processes, cell division, or cell motility. • Discuss the connection between deranged cell signaling pathways and diseases, such as cancer. • Propose specific interventional approaches for diseases based on knowledge of cell signaling pathways.	• Essay covering the key points of the learning outcomes. • Group presentation on the pathway of interest	• Use Supporting Material S3. • 30 minutes: The instructor introduces the students to the RGD and NCBI’s Gene database through in-class demonstration. It is helpful to use the same pathway assigned in the reading (above) to pinpoint the key proteins and their reported functions (http://rgd.mcw.edu/wg/home/pathway2/molecular-pathways2). 30 minutes: Students explore a pathway of interest on RGD. They then retrieve information from the Gene database (http://www.ncbi.nlm.nih.gov/gene/?term=) on the function of specific genes in the pathway. • 30-minutes: instructor-led discussion on the different stages of signal transduction including the associated proteins and their enzymatic activities. • 30-minutes: instructor-led session discusses the style of writing the introduction section of a scientific paper (see “Notes for instructors” in the introductory section of S3.) • 30 minutes: Students identify diseases associated with specific genes through OMIM (http://www.ncbi.nlm.nih.gov/omim).

**Module 2 (Two 3-hour Lab Sessions, 6 hours total)**			
Although many students will have personal laptops, some rely on tablets, which are not suitable for this activity. Work with IT to install software on the students’ computers or use a computer lab. Groups of three to five students are ideal for this activity.	Students will be able to: • Use software to analyze and interpret gene expression data. • Use appropriate statistical method for hypothesis testing • Manipulate large datasets with software	Students answer specific short answer questions designed to reveal proficiency in the execution of the different steps of the lab. Alternatively, students could develop a lab report with the key elements of a research paper (abstract, introduction, materials and methods, results, discussion and conclusion).	• Use Supporting Material S4. • 30 Minutes: Instructor demonstration of relevant NCBI database (https://www.ncbi.nlm.nih.gov/geo/) and online data analysis tools. Note that data can be analyzed through GEO DataSets analysis tools or through GEO2R through this site. • Assign GEO tutorial that includes a video from the NCBI website (https://www.ncbi.nlm.nih.gov/geo/info/geo2r.html) • 1 hour: Students retrieve a dataset selected by the instructor from GEO (http://www.ncbi.nlm.nih.gov/geo/). • Students analyze the data with online tools with the Geo Database and respond to questions in S4. • 1 hour and as an assignment: with geWorkbench. • 2 hours: Students identify the biological functions of genes showing significant alterations in expression levels with • Database for Annotation, Visualization and Integrated Discovery (DAVID) https://david.ncifcrf.gov/home.jsp • 45 minutes: Discuss the structure of the results section of a scientific paper with students. • Final reports that include the background, results, and conclusion sections.

## Learning Goal(s)

- Students will understand the fundamentals of cell signaling and its relationship to human diseases.
- Students will gain insight into the process of scientific investigations and practice scientific communication.
- Students will gain experience in the use of biological databases and in the analysis of large datasets.

## Learning Objective(s)

Students will be able to:

- Explain the hierarchical organization of signal transduction pathways.
- Explain the role of enzymes in signal propagation and amplification.
- Recognize the centrality of signaling pathways in cellular processes, such as metabolism, cell division, or cell motility.
- Rationalize the etiologic basis of disease in terms of deranged signaling pathways.
- Use software to analyze and interpret gene expression data.
- Use an appropriate statistical method for hypotheses testing.
- Produce reports that are written in scientific style.
