# Supplementary material for: Investigating Cell Signaling with Gene Expression Datasets
Source: CourseSource. Author manuscript; Available in PMC 2020 Aug 26. (PMC7449260; doi:10.24918/cs.2019.1)
Supplement: S1 [file NIHMS1030899-supplement-S1.docx]

**S1: Foundational Knowledge**

**Note to instructors**

These ideas are introduced at the beginning of the semester to lay the foundation for inquiry-based learning. The fine technical details need not be mastered but the students should become familiar with the applications of the techniques mentioned. Further, short narrated videos are very effective in introducing students to the subject matter prior to delving into research papers because the presented data become more meaningful if students understand their genesis. Making connections to diseases, or other human endeavors, motivates students to ask questions and seek more information. Note that the materials below serve to supplement or extend what is already in the textbook and we do not wish to introduce brand new information at this stage.

1. Supplement the microscopy sections of the textbook with the following links:

Fluorescent proteins: <https://www.youtube.com/watch?v=x5ox71qIa-0>

Advances in intracellular imaging: <https://www.ibiology.org/?s=lippincott>

1. Supplement the cellular fractionation section of the textbook with this or similar link that discusses identification of a receptor for animal tissues:

<https://hms.harvard.edu/news/recipe-success>

This very short video summarizes the following paper published in PNAS. The goal is to generate interest without delving too much into the technical aspects of the paper. However, reading the abstract allows students the opportunity to make connections between the textbook description of organelles, the ER in this case, and human diseases.

Alon A, Schmidt HR, Wood MD, Sahn JJ, Martin SF, Kruse AC. Identification of

the gene that codes for the σ(2) receptor. Proc Natl Acad Sci U S A. 2017 Jul

3;114(27):7160-7165. doi: 10.1073/PNAS.1705154114. Epub 2017 May 30. PubMed PMID: 28559337; PubMed Central PMCID: PMC5502638.

1. Supplement the key technique section of the text Determining the Chemical Fingerprint of a Cell Using Mass Spectrometry with this link. Organic chemistry is a co-requisite for our course and thus, students may have some knowledge of chemical characterization techniques.

<https://www.ibiology.org/techniques/mass-spectrometry/>

1. Culture of animal cells:

<http://www.ncbi.nlm.nih.gov/books/NBK21682/>
