# Supplementary material for: Investigating Cell Signaling with Gene Expression Datasets
Source: CourseSource. Author manuscript; Available in PMC 2020 Aug 26. (PMC7449260; doi:10.24918/cs.2019.1)
Supplement: S2 [file NIHMS1030899-supplement-S2.docx]

**S2: Signaling Introductory Lecture Outline**

Ligand-receptor interactions outputs: Membrane receptors respond to ligand binding in 3 general ways. (i) Receptors classified as 7 transmembrane-spanning receptors (7TMS) also called G protein-coupled receptors (GPCRs) respond by stimulating enzymes to produce second messengers, such as cyclic adenosine monophosphate (cAMP). Adrenaline receptors function in this manner. (ii) Growth factor receptors undergo changes, usually phosphorylation, in their intracellular domains to create docking sites for intracellular signaling proteins. The insulin receptor functions in this manner. (iii) Ligand gated ion-channels respond by allowing the flow of ions down the concentration gradient. Neurotransmitter receptors use this mechanism

Signal transduction: Signal transduction pathways are series of proteins arranged in a hierarchical manner. The proteins proximal to the receptor become active first and they in turn activate the next protein in the pathway. The signal is transmitted sequentially to effectors of the pathway. Signal transduction is accompanied by amplification at each step. For example, in the adrenaline signaling pathway, a trimeric G-protein is activated first and it in turn activates adenylyl cyclase. Adenylyl cyclase produces many molecules of cAMP, which then activates other signal transduction proteins, such as protein kinase A (PKA).

Response: The signal transduction pathway culminates in the activation of effector proteins. In this example, protein kinase A phosphorylates glycogen phosphorylase kinase and leads to the activation of glycogen phosphorylase. Glycogen phosphorylase hydrolyses glycogen to release glucose that will be metabolized to provide cellular energy.

Role of protein phosphorylation: Many signal transducers are protein kinases. Intracellular kinases are usually serine/threonine kinases while growth factor receptors are tyrosine kinases referred to as receptor tyrosine kinases (RTKs). The action of kinases is reversed by phosphatases. Most kinases act on only a few substrates but the phosphatases are more general. The targets of the kinases include transcription factors, ion channels, metabolic regulators, and cytoskeleton components. Depending on the target protein, phosphorylation can lead to activation, inactivation, degradation or stabilization of the protein.

Signal Termination: Finally the signal is terminated through the reversal of posttranslational modifications, degradation of primary and second messengers, and removal of receptors from the cell surface through endocytosis.

Other Resources

Note that the video resources below are amenable to a flipped classroom teaching model. In our case, we find it effective to post a video on our learning management system (Blackboard) with Panopto (<https://www.panopto.com/>), which allows for annotations to be added to the video. As students watch the video, they are prompted to answer questions in a quiz format. Their responses are later discussed in class and expanded upon.

Video Presentations:

Seven Transmembrane Receptors, G protein coupled receptor kinases, and Beta-arrestins (Robert Lefkowitz): <https://www.ibiology.org/cell-biology/g-protein-coupled-receptors/>

Protein Kinases: Structure, Function, and Regulation (Susan Taylor): <https://www.ibiology.org/biochemistry/protein-kinase/>

Overview summary:

Scitable by Nature (Cell Signaling): <https://www.nature.com/scitable/topicpage/cell-signaling-14047077>
