# Supplementary material for: Investigating Cell Signaling with Gene Expression Datasets
Source: CourseSource. Author manuscript; Available in PMC 2020 Aug 26. (PMC7449260; doi:10.24918/cs.2019.1)
Supplement: S3 [file NIHMS1030899-supplement-S3.docx]

**S3: Cell Signaling Overview**

**Learning Goals**

1. Students will understand the fundamentals of cell signaling and its relationship to human diseases.
2. Students will gain insight into the process of scientific investigations and practice scientific communication.

**Learning Outcomes**

The students will be able to:

1. Identify or describe the different stages of cell signaling.
2. Discuss the organization of different signal transduction pathways.
3. Explain how different enzymes achieve amplification of the signal during transduction.
4. Correlate signaling pathways to different cellular responses.
5. Discuss the connection between deranged cell signaling pathways and diseases, such as cancer.

**Role of Cell Signaling in Cell Proliferation and Viability**

**Summary**

Polar molecules, such as peptide hormones, cytokines, and amino-acid derived signaling molecules function through cell surface receptors. The receptors generally fall under several classes including G protein-coupled receptors (GPCRs), ion-channel receptors, tyrosine-kinase linked receptors, and receptors with intrinsic enzyme activity.

Receptors with intrinsic enzymatic activity contain a ligand-binding site in the extracellular domain and an enzymatic activity-containing domain in the intracellular domain. Receptor tyrosine kinases (RTKs), such as the epidermal growth factor receptor (EGFR) are good examples of this class. Binding of a ligand stimulates receptor aggregation and cross-phosphorylation of the intracellular domains, which then creates binding sites for other signaling molecules. In the case of EGFR, the protein GRB2, an adaptor protein is recruited to the activated receptor and it, in turn, recruits SOS, an activator of the small G protein Ras. Activated Ras then stimulates the mitogen activated protein kinase pathway (MAPK). Human EGFR (HER2) is the therapeutic target for monoclonal antibodies used in the treatment of breast cancer, for example, trastuzumab (Herceptin), and the kinase inhibitor lapatinib (tykerb). Note that one oncogenic transformation in breast cancer involves the amplification of HER2 gene. Ras is also one of the most commonly mutated genes in cancers.

***Notes for instructors***

The goal of this activity is to provide students with an understanding the hierarchical organization of signal transduction pathways and to make connections between cell biology and society. All upper-level cell biology textbooks will have a section on growth factor signaling and it is recommended that just the relevant section, which is just three pages in the Becker’s World of the Cell be assigned to the students. This can be covered in a 50-minute lecture. In the next stage, students investigate the pathway of interest in detail in a computer lab session. A link to a free textbook can be found at the end of this page.

***Students’ instructions***

The introduction section of a research paper provides the background to the subject matter citing the most current or impactful publications. It indicates the relevance and importance of the subject matter. The gaps or areas needing further study are clearly stated. A final paragraph alludes to the hypothesis that was tested. A final sentence summarizes the major findings.

**Notes for instructors:** It possible to illustrate the above organization of the introduction section of a cell and molecular biology paper by asking students to identify the above components and paraphrase the concepts within a 20-minute class discussion. The students need to appreciate not only the scientific content but also the style of scientific communication. For example, and without being too prescriptive, the students could be asked open the AKT1 object from the pathway below and then link out to PubMed to retrieve a short article, such as “Baba T, Kobayashi H, Kawasaki H, Mineki R, Naito H, Ohmori D. Glyceraldehyde-3-phosphate dehydrogenase interacts with phosphorylated Akt resulting from increased blood glucose in rat cardiac muscle. FEBS Lett. 2010 Jul 2;584(13):2796-800. doi: 10.1016/j.febslet.2010.05.015. Epub 2010 May 17. PubMed PMID: 20488185.”. The one-page introduction of the article can be discussed. Further, the already introduced techniques, such as cell culture and mass spectrometry can be highlighted.

Also, note that learning to use scientific databases and literature is a fundamental skill of modern science, just like acquiring expertise in experimental techniques. Students at this level should be introduced to both aspects in line with the recommendations of Vision & Change.

(1) Based on the reading of the article below, provide a brief overview of the pathways of interest:

The example given here is on PI3K/AKT.

<http://rgd.mcw.edu/rgdweb/pathway/pathwayRecord.html?processType=view&species=Rat&acc_id=PW:0000232>


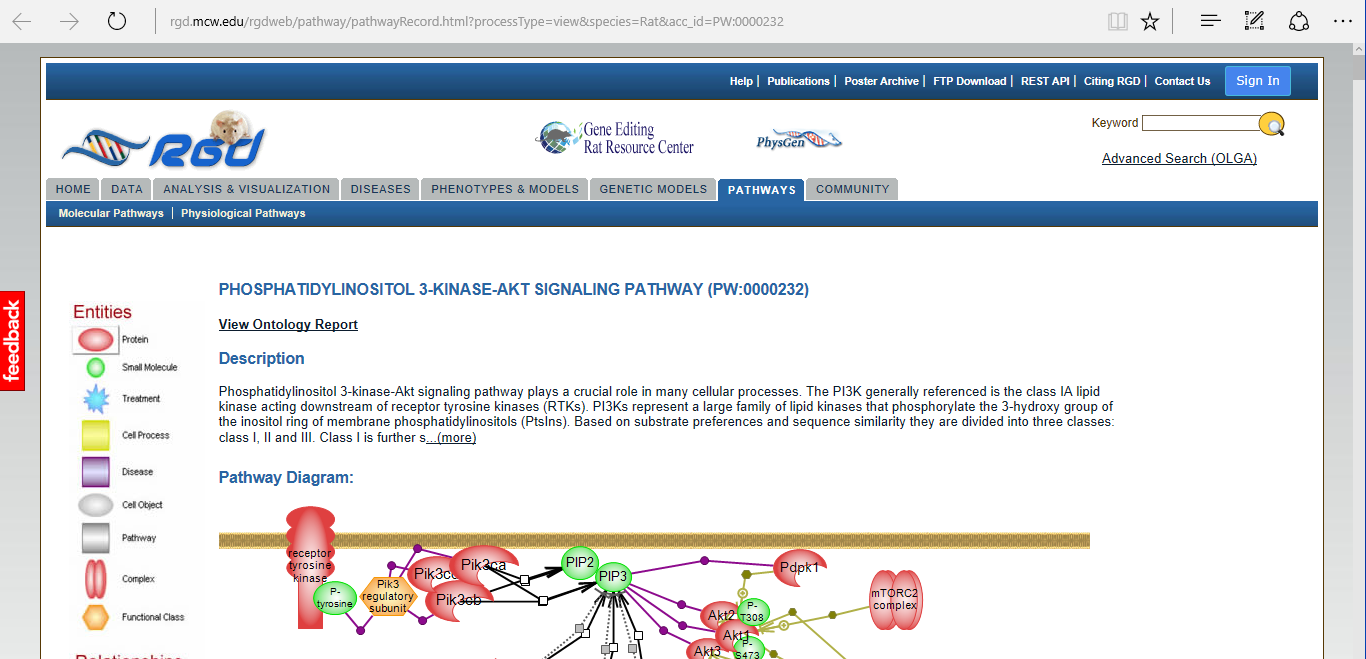


***Answer:***

*The PI3K/AKT signaling pathway is activated by receptors with intrinsic enzymatic activity. Activation of insulin receptor (InsR) in addition to activating the MAPK pathway above also stimulates glucose uptake and metabolism as well as cell growth and survival through the PI3K-AKT pathway. Phosphorylated motifs in the intracellular domains of RTKs are recognized by SH2 (Src homology 2) domains of intracellular signaling proteins. In this case, PI3K is recruited to the plasma membrane through the binding of regulatory domain. PI3K phosphorylates phosphatidylinositol-4,5-bisphophate (PIP2) to generate PIP3. PIP3 acts as the docking site for the plekstrin homology domains of AKT and its activator kinase PDK1 (PDPK1) thereby leading to the activation of AKT. PKB/AKT is activated by phosphorylation at threonine 308 (T308) in the activation loop. Full activation requires the phosphorylation of S473 in the hydrophobic motif.*

 (2) The signal for this pathway flows from the receptor to effectors in the cytoplasm and the nucleus. Cytoplasmic responses include the increased uptake of glucose while nuclear effects promote cell survival. Open the links for selected gene products in the pathway and write short summaries on their function. Note that linking out to the NCBI Gene database will lead to a summary of the gene function and other other links to molecular information on the gene.


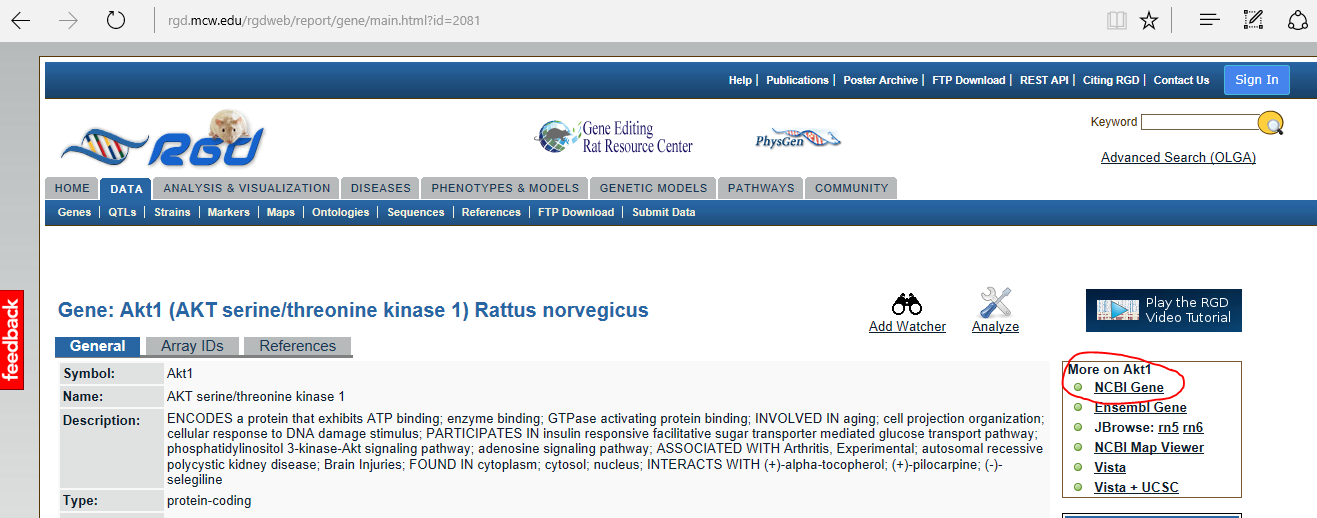


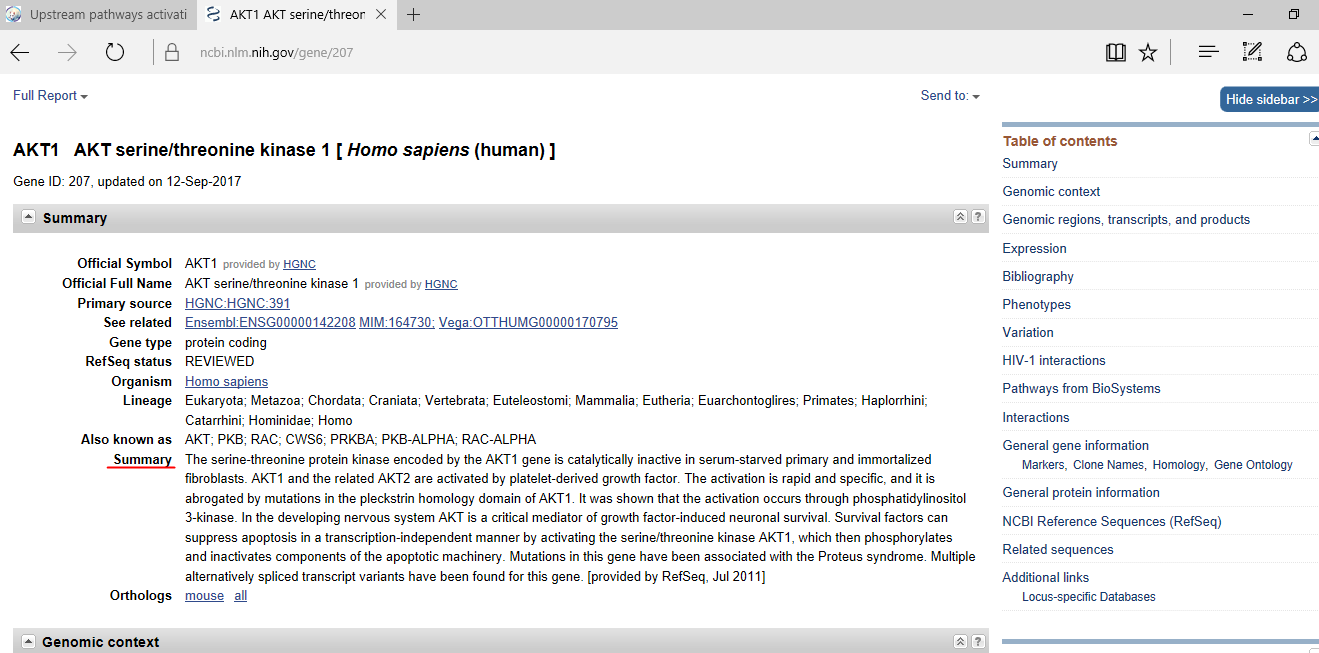


***Answer:***

*AKT1 functions downstream of phosphatidylinositol 3'-kinase, which in turn acts downstream of receptor tyrosine kinases such as insulin receptor and growth factor receptors. Note that PI 3-kinase phosphorylates inositol lipids in the intracellular leaflet of the bilayer and thereby creates docking sites for the PH domain of AKT. AKT is recruited to the plasma membrane where it is phosphorylated at the activation loop. This phosphorylation of the activation loop and the HM leads to a change in conformation from disordered to ordered form. The change allows access to the enzyme's active site.*

*The activated AKT dissociates from the membrane and targets other proteins in the cytoplasm and the nucleus. AKT substrates have a conserved motif of -RXRXXSTB-, where X is any amino acid and B is a bulky hydrophobic amino acid. The effectors of AKT regulate metabolism, cell growth proliferation, cell survival and migration.*

(3) Propose a hypothesis on the role of any of the above kinases in the proliferation and viability of U-937 cells.

***Answer:***

*Hypothesis: Based on the known functions of AKT, it can be hypothesized that the PI3K/AKT pathway is dysregulated in U937 cells leading to uncontrolled cell growth and proliferation.*

1. From the “AKT1 gene” page under related information, open the protein link and then identify the functions associated with each domain of the protein.

Example of expected response:
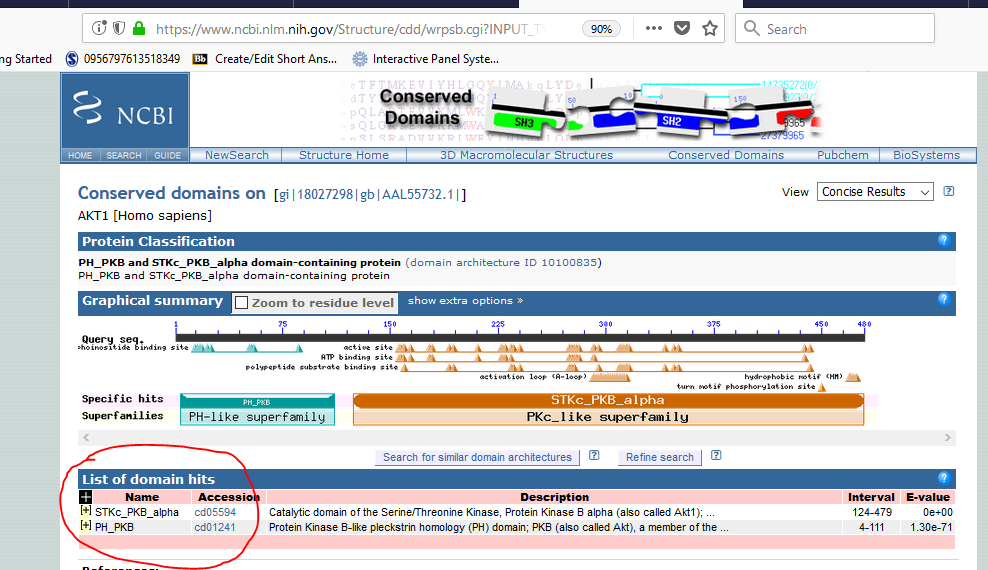


The two domains of AKT are identified as the N-terminal pleckstrin homology (PH) domain and the central Ser/Thr kinase domain, which is followed by the C-terminal hydrophobic motif (HM). The PH domain binds to inositol lipids in the intracellular leaflet of the bilayer. The phosphorylation of the activation loop and the HM leads to a change in conformation from disordered to ordered form. This change allows access to the enzyme's active site. The central Ser/Thr kinase domain bears the enzyme’s active site.

1. How are AGC kinases activated? (hint: click on the turn motif phosphorylation site under question 4 and then follow up on the literature citation).

**A*nswer****:* AGC kinases are activated through the phosphorylation of the activation loop and the HM. This causes conformational changes that make the catalytic site accessible to substrates. Note that there are enzyme specific-regulatory mechanisms in addition to phosphorylation. For example, protein kinase A (PKA) is heterotetramer of two cAMP binding regulatory subunits and two catalytic subunits. The catalytic subunits exist in phosphorylated form but the activity is blocked by the bound regulatory subunits. Binding of cAMP to the regulatory subunits causes their release from the catalytic subunits and activation of the latter.

(6) Identify the cellular pathways in which AKT1 is involved.

***Answer:***

AKT1 functions downstream of phosphatidylinositol 3'-kinase, which in turn acts downstream of receptor tyrosine kinases such as insulin receptor and growth factor receptors. The PI3K/AKT pathway mediates the functions of some growth factors, such as insulin and cytokines

(7) From the “AKT1 gene” page, follow the OMIM link, and identify some diseases associated with its mutations.

<https://www.ncbi.nlm.nih.gov/omim?LinkName=gene_omim&from_uid=207>

***Answer:***

*Most of the diseases associated with mutations of AKT relate to defective cell growth, proliferation or migration. This is consistent with the pathways in which it is involved.*

**References**

<https://cnx.org/contents/GFy_h8cu@11.2:H4oMpCSi@9/Signaling-Molecules-and-Cellul>

Name: Biology

ID: 185cbf87-c72e-48f5-b51e-f14f21b5eabd@11.2

Language: English (English)

Summary: Biology is designed for multi-semester biology courses for science majors. It is grounded on an evolutionary basis and includes exciting features that highlight careers in the biological sciences and everyday applications of the concepts at hand. To meet the needs of today’s instructors and students, some content has been strategically condensed while maintaining the overall scope and coverage of traditional texts for this course. Instructors can customize the book, adapting it to the approach that works best in their classroom. Biology also includes an innovative art program that incorporates critical thinking and clicker questions to help students understand—and apply—key concepts.

Subjects:Science and Technology

License: Creative Commons Attribution License (by 4.0)

Authors: OpenStax

Copyright Holders: Rice University

Publishers: OpenStaxOpenStax Biology

Latest Version: 11.2

First Publication Date: ‎Aug‎ ‎22‎, ‎2012

Latest Revision: Jun‎ ‎1‎, ‎2018

Last Edited By: Ryan Stickney
