# Supplementary material for: Investigating Cell Signaling with Gene Expression Datasets
Source: CourseSource. Author manuscript; Available in PMC 2020 Aug 26. (PMC7449260; doi:10.24918/cs.2019.1)

**S4: Functional Genomics Lab**

**Introduction**

High through-put molecular biology techniques entail highly parallelized experiments to simultaneously measure the levels of different molecules such as RNA, proteins, and metabolites. The terms used for such experiments include the suffix “-omics”. The measurement of all expressed transcripts in an experimental system is called transcriptomics, that of proteins is called proteomics, and measurement of all metabolites is called metabolomics.

Microarray technology, and more recently RNA sequencing (RNA-seq), have revolutionized molecular biology and biomedical research in that they allow for the measurement of the levels of a multitude of transcripts simultaneously. Given this, the data generated has high dimensionality and cannot be reasonably analyzed or understood with manual techniques.

Proteomics, microarray, and RNA-seq studies, collectively called functional genomics, aim to reveal global gene expression patterns in a system under study. Gene expression patterns are correlated with phenotypes of interest. For example, in experiments designed to understand the molecular basis for cancer, RNA isolated from a tumor tissue is compared to that isolated from a normal tissue. The differences in expression levels of genes in the two samples provide an insight to the molecular changes leading to the disease, the molecular etiology, and can identify genes for use in diagnosis or prognosis.

As part of the publication process of research data, investigators also deposit the raw data from functional genomics studies in publicly accessible databases such as GEO (<http://www.ncbi.nlm.nih.gov/gds/?term=>). The data can then be used to test and generate different hypotheses beyond the scope of the original study.

**Notes for instructors:** It is helpful to provide some background information on microarray technology and there are many resources of the Web. However, these two are particularly pertinent to a data analysis pipeline.

How to Analyze DNA Microarray Data

<https://www.hhmi.org/biointeractive/how-analyze-dna-microarray-data>

**AND**

Joseph DeRisi, PhD., UC San Francisco

Malaria: The Disease and Parasite Plasmodium falciparum

<https://www.ibiology.org/microbiology/plasmodium-falciparum/#part-2>

NCBI Tutorials (Please search GEO and assign the video)

https://www.ncbi.nlm.nih.gov/home/tutorials/

**Problem Definition**

In research and clinical settings, it is often necessary to distinguish two samples based on their gene expression profiles. For example, cancer of a particular tissue was thought to be a single disease, but now, for example, we know that there are 4 major types of lung cancer, each with its own set of differentially expressed genes. Based on the molecular differences, therapies can be better targeted to the type of tumor, i.e., in an approach referred to as pharmacogenomics or precision medicine. To discover the differences that distinguish different tumors, gene expression profiles comparing normal tissue to disease tissue are obtained with functional genomics techniques. Therapies are then selected based on the genetic profile of the tumor.

Given that the data are accessible, other investigators can test new hypotheses or ask new questions about the experimental condition.

**Learning Goals**

1. Students will gain insight into the process of scientific investigations and practice scientific communication.
2. Students will gain experience in the use of biological databases and in the analysis of large datasets.

**Learning Outcomes**

Students will be able to:

1. Use software to analyze and interpret gene expression data.
2. Use appropriate statistical method for hypothesis testing.
3. Manipulate large datasets with software.

**Procedure**

1. Go to Geo DataSet database and retrieve data from the paper by Zhang et al., 2013 reporting on effect of bone marrow microenvironment on imatinib treated CML cells (<http://www.ncbi.nlm.nih.gov/sites/GDSbrowser?acc=GDS4756>). The data will be under DataSet full SOFT file. Save this file in a folder, as it will be needed later in this protocol.
2. Identify the hypothesis being tested in this experiment by briefly reading the abstract in PubMed database.

**Notes for Instructors:** While a hypothesis is not explicitly stated in most molecular and cell biology papers, it is nevertheless implicit in the publications and the goal of this questions is to prompt the students to discern the impetus of the study. The students therefore read the abstract more deeply in a search for a hypothesis. This step can be used to examine the format of abstracts in scientific papers. The guidance for students and additional notes for instructors for the Introduction section are provided in Supporting File S3. Here, based on time constraints, the instructor could help students deconstruct the abstract as follows:

- 1. An introductory sentence or sentences (Sentences 1 and 2: Whereas TKIs are effective in treating chronic myeloid leukemia (CML), their use is associated with persistence of leukemia stem cells followed by relapse.)
  2. A statement of the goal (see hypothesis below)
  3. Methodology or Experimental system (treatment of co-cultures of human bone-marrow mesenchymal cells with CML progenitor cells)
  4. Summary of the major findings:
     1. Human bone marrow mesenchymal stromal cells (MSCs) inhibit apoptosis of chronic myeloid leukemia cells that is induced by tyrosine kinase inhibitor (TKI) (supported by figures 1 and 2, mention but no need to explain methodology).
     2. N-cadherin receptor is involved in the protection of CML progenitors by MSCs (supported by figure 3).
     3. N-cadherin mediated adhesion of CML progenitors to MSCs is involved in the nuclear translocation of beta-catenin (supported by figure 4).
     4. N-cadherin mediated adhesion of CML progenitors to MSCs is involved in transcriptional activity of beta-catenin (supported by figure 5, the microarray experiment).
     5. Exogenous Wnt mediates beta-catenin signaling (supported by figure 6)
  5. A final concluding statement that provides an interpretation to the results (Last two sentences; The study implicates two signaling pathways (N-cadherin and Wnt-beta-catenin pathways) in TKI resistance in CML)

The data for this lab are from a study published in Blood by Zhang et al. (2013). The study was motivated by the observation that while tyrosine kinase inhibitors (TKIs) are effective in treating chronic myeloid leukemia and are cytotoxic to BCR-Abl oncogene carrying leukemia stem cells (LSCs), patients treated with the inhibitors nevertheless relapse, which indicates the persistence of LSCs in the bone marrow.

**Answer:** A good hypothesis statement has three components. Firstly, it states what will be changed (the independent variable) during the study. Secondly, it predicts the outcome of the change (the effect on the dependent variable) and, thirdly, it provides a rationale for why the investigator believes that the predicted results will occur. In this case, empirical evidence or literature indicate that although TKIs exhibit cytotoxicity towards CML cells by inhibiting the BCR-Abl kinase, patients nevertheless go on to relapse. The hypothesis then addresses the cause of preservation of LSCs during treatment. The hypothesis for this study therefore posits that the bone marrow microenvironment signals to LSCs causing changes in gene expression patterns and thereby blocking apoptosis induced by TKIs.

**Prediction:** CML cells cultured in the presence of bone marrow mesenchymal stromal cells will be more resistant to imatinib because they experience growth stimulatory signals that bypass the BCR-Abl oncogene.

**Critical Experiment:** A transcriptomics experiment was used to identify the cell signaling pathway that confer cell survival ability to CML cells in the presence of imatinib.

1. What is the target of imatinib?

(<http://www.drugbank.ca/drugs/DB00619>)

**Notes to instructors:** One major goal of this lab is to inform students of the many online resources supporting current biomedical research. The DrugBank is comprehensive repository of approved drugs and their targets and it provides comprehensive ancillary information on the drugs including toxicological profiles and mechanisms of function. In this step, the students retrieve information on the molecular mechanisms of function of imatinib.

**Answer:** Imatinib is an example of a rationally designed drug for cancer treatment because its development involved identifying molecules that specifically inhibit a mutated protein in cancer cells. Whereas drugs are designed to target specific molecules and imatinib was discovered as a specific inhibitor of the BCR-Abl oncogene, subsequent research has demonstrated that it also inhibits other tyrosine kinases. Off-target effects can lead to toxicity or expanded use of a compound.

1. Is imatinib a molecular therapy? Explain. (<https://www.pennmedicine.org/cancer/navigating-cancer-care/treatment-types/targeted-therapy>)

**Notes for instructors:** During the course of this lab, students are expected to appreciate the role of molecular structure determination in the development of drugs for various diseases, in this case cancer. Cancer cells are associated with specific molecular changes, oncogenes, that lock them in the cell cycle inappropriately. To best communicate the application of science in society, students should be directed to authoritative sources of information. Further, it may be necessary to provide additional background to the students.

**Answer:** Given that imatinib targets a gene that is mutated in leukemia, it is considered a molecular therapy. In particular, recent research has determined that cancer is not just one disease but many diseases each with its own molecular etiology. Based on this, therapies can be tailored for individual patients based on the molecular phenotypes of the tumors. Imatinib is suitable for cancers caused by BCR-Abl translocation or Philadelphia chromosome.

1. The data presented can be analyzed in different ways. Under the Data Analysis Tools tab, identify at least two combinations of control and treatment groups.

**Notes for instructors:** The instructor will need to familiarize herself/himself with the database beforehand, which will take about 30 minutes (<https://www.ncbi.nlm.nih.gov/geo/>). The instructions are available on the website.


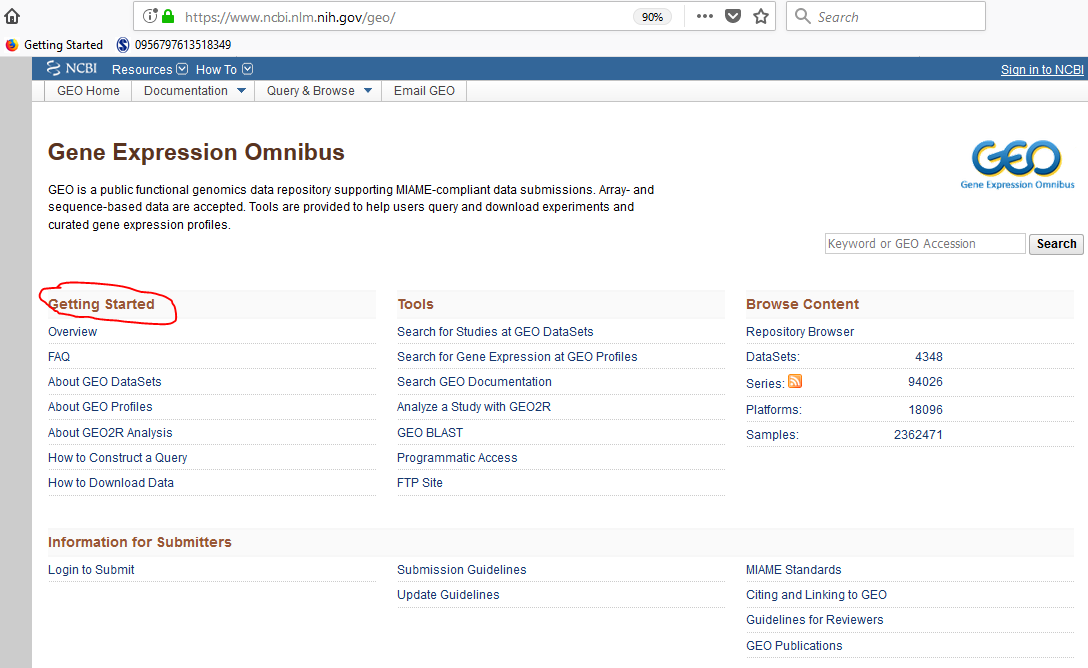


The study contains eight different sample groups as follows (see <https://www.ncbi.nlm.nih.gov/sites/GDSbrowser?acc=GDS4756#details>):

- - 1. Peripheral blood cells alone left untreated or treated with imatinib
    2. Bone marrow cells alone left untreated or treated with imatinib
    3. Co-cultures of peripheral blood and mesenchymal stromal cells left untreated or treated with imatinib
    4. Co-cultures bone marrow and mesenchymal stromal cells left untreated or treated with imatinib

The authors report in the paper that for the purposes of statistical analysis all data for the different tissues were combined into a set of untreated and treated samples. The different tissues (peripheral blood or bone marrow) were not separated. However, given that it not necessary for the students to read the full article, the students should identify the most reasonable groups of samples to compare and provide an appropriate justification.

The students in this course more than likely have been introduced to *t* tests in other courses. However, this is the opportunity to consolidate their understanding of how statistics are utilized in hypothesis testing. A good resource for teaching students the basics of hypothesis testing using *t* tests is provided by the University of Maryland at: <http://mathbench.umd.edu/modules/statistical-tests_t-tests/page01.htm>. The authors have developed assessments that can be requested.

1. Still under data analysis tools tab, compare two samples at the significance levels of 0.1, 0.05, and 0.01.
   1. What does the level of significance indicate?

**Answer:** It indicates the probability of obtaining the result seen purely by chance. A *p* value of <0.05 is routinely used in biological literature and at this threshold there is a 5% chance of erroneously rejecting the null hypothesis. Please note that with high-throughput experiments, such as microarray experiments, the number of variables (genes) is very large. These datasets, therefore, should be analyzed using procedures developed for correcting for errors associated with multiple testing.

- 1. Explain the differences, if any, in results obtained (number of records retrieved) at different thresholds.

**Answer:** With a larger *p* value (for example, 0.1) you expect to obtain more hits. Many of them will be false positives. A more stringent *p* value (0.01) produces fewer hits, but you may also lose some data as false negatives. Thus, the risk of obtaining a type I error is balanced with one for obtaining a type II error. Routinely, the experiments are followed by more focused experiments, such as the use of quantitative real-time polymerase chain reaction (RT-qPCR) to validate the results of microarray experiments. A similar problem is encountered with data obtained with yeast 2-hybrid assays. A large number of hits from these assays are false positives and thus, the experiments are verified with additional experiments, such co-immunoprecipitation-immunoblot experiments.

1. Compare the data using a clustering method.
   1. What advantages does clustering offer to the analysis of high-dimensional data? (<http://www.ncbi.nlm.nih.gov/geo/info/cluster.html>)

**Feedback to students:** Clustering is a data mining technique aimed at identifying patterns in datasets. It is exploratory data analysis aimed at gaining an overview of the distribution of expression patterns and, hence, identifying groups of genes or clusters that merit further analysis.

- 1. Under (5) an experimental and a control group were selected. Identify some differentially expressed genes.

**Notes for instructors:** This step is intended to help students gain knowledge on how to prioritize information from high content data and to navigate the different molecular databases. Analysis of two samples, untreated and imatinib treated peripheral blood samples at a *p* value of 0.01 yields 248 differentially expressed genes. Based on the student's hypothesis, they can be directed to identify genes associated with apoptosis or cell growth and proliferation from the retrieved dataset. For easier viewing and retrieval, students can be asked to set the "items per page" shown under "Display Settings" to 500. The instructor could download a list of apoptosis associated genes from the GSEA website (<http://software.broadinstitute.org/gsea/msigdb/cards/KEGG_APOPTOSIS.html>). From this site, the KEGG pathway map can be displayed and discussed in the context of the pathway shown in the textbook, for example, Becker's World of the Cell by Jeff Hardin and Gregory Bertoni, 9e, pages 741-743.


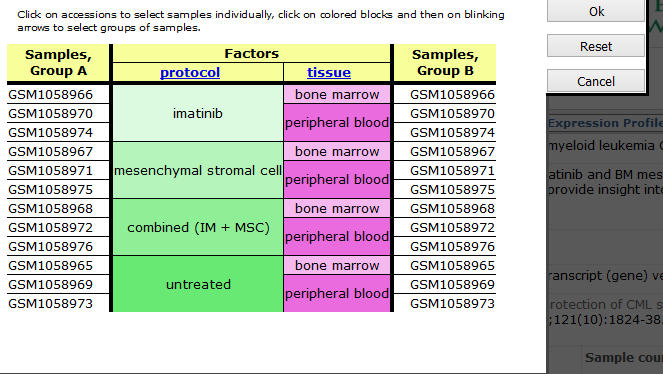


1. Unzip the DataSet full SOFT file.

**Notes for instructors:** These are large files and downloaded as compressed files. Most modern computers will have preinstalled software for extracting compressed files. Alternatively, there are many free programs for extracting files.

1. Open the file with geWorkbench for analysis. Note that an annotation file is needed to view annotations and higher order analysis of expression profiles.

**Notes for instructors:** The annotation file is supplied by the manufacturer of the microarray, in this case Affymetrix, and free registration may be required.

Under sample subsets, click on the platform (GPL6244) and in the next screen click on the Affymetrix technical support link.

##
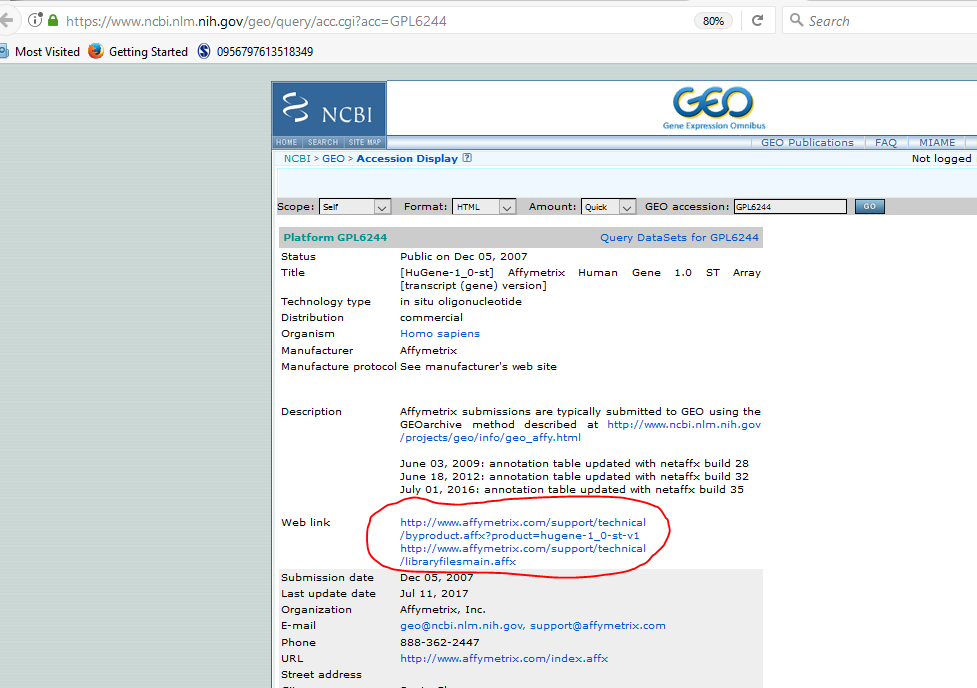


1. Experiment with different *p*-values and export the data to local computer.

**Notes for instructors:** This step is similar to step 6 above except now students have more control over data analysis and could use more techniques, such as ANOVA. It is necessary for the instructor to first follow the online tutorial at the geWorkbench website (<http://wiki.c2b2.columbia.edu/workbench/index.php/Home>). Below are sample outputs from the software.

**Answer: Upon successful upload of the data you should see:**


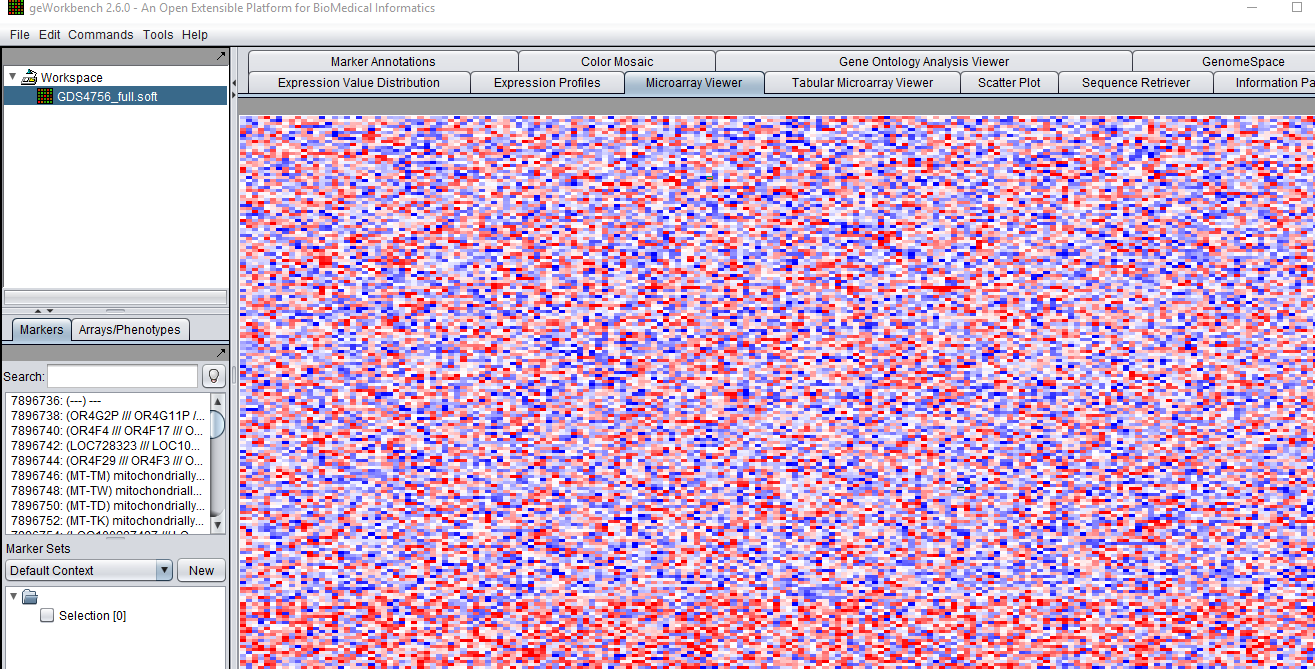


**Select the samples to analyze:**


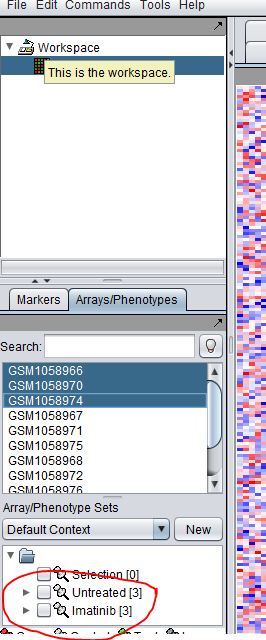


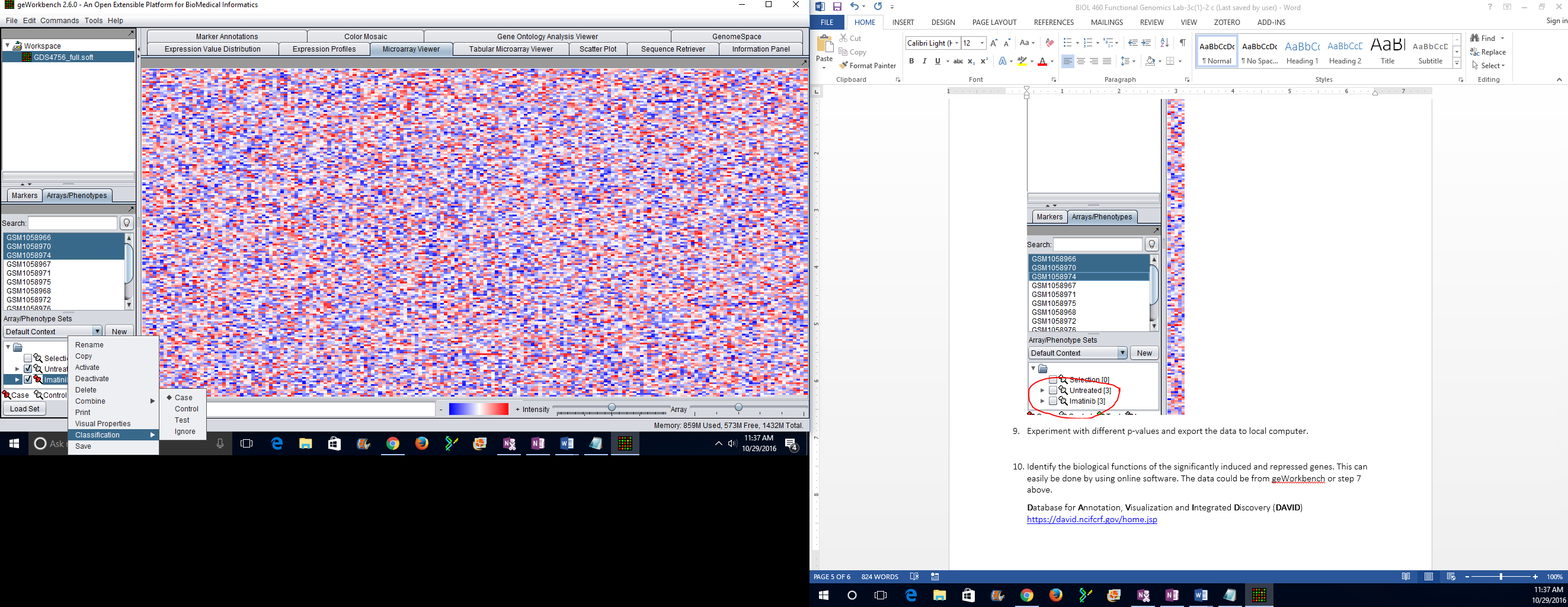


1. Experiment with different p-values and export the data to local computer.

**Perform the *t* test**


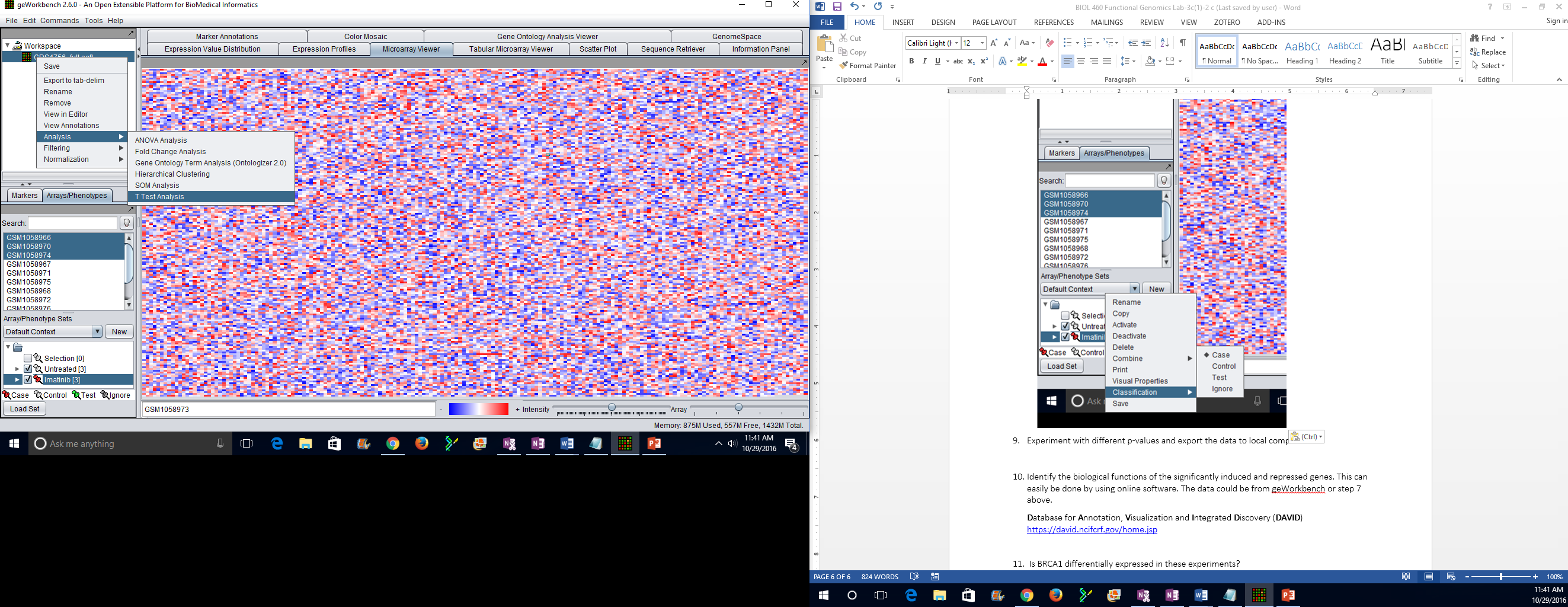


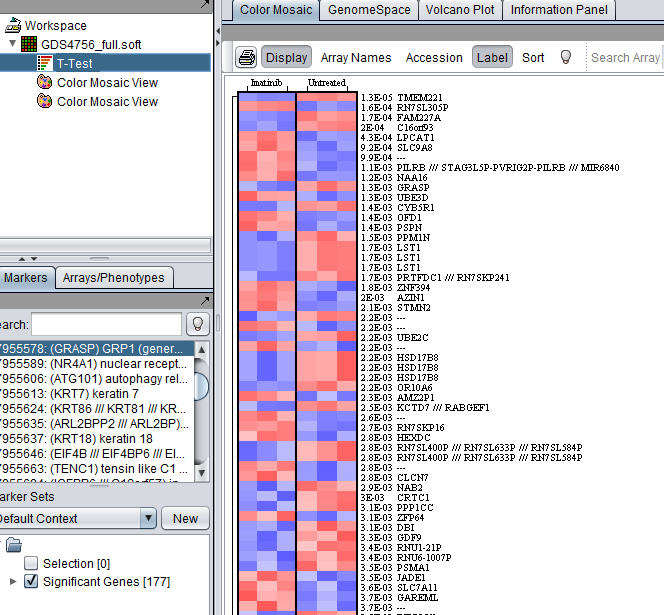


**Answer:**

The genes showing significant differences in expression levels are exported as a .csv file to a local computer for further analysis with DAVID.

Data visualization with Volcano Plot


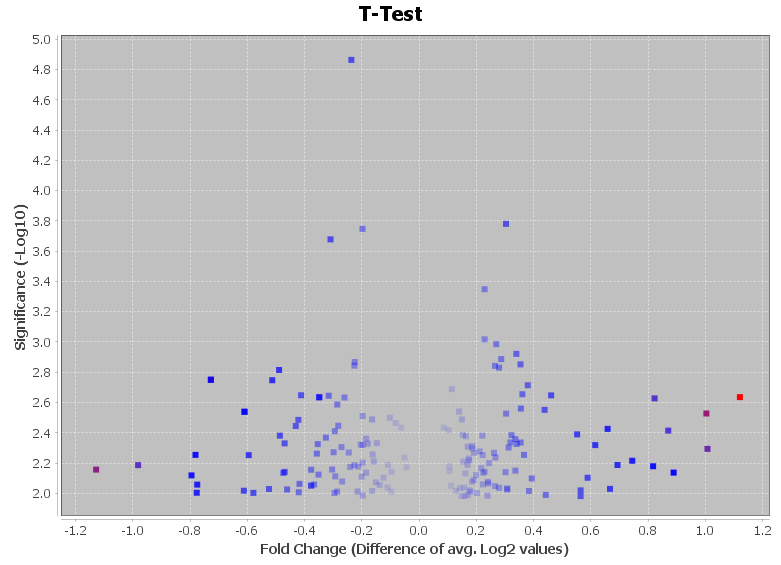


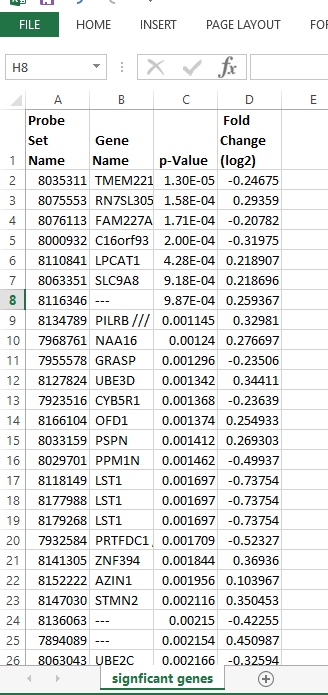


1. Identify the biological functions of the significantly induced and repressed genes. This can easily be done by using online software. The data could be from geWorkbench or step 7 above.

**D**atabase for **A**nnotation, **V**isualization and **I**ntegrated **D**iscovery (**DAVID**) <https://david.ncifcrf.gov/home.jsp>

**Notes for instructors:** Only the symbols for differentially expressed genes are uploaded to DAVID. The symbols are simply copied and pasted into the text box. Again, it is important for the instructor to become familiar with the website beforehand. We prefer to conduct a demonstration for the students to help them identify the different features needed to efficiently analyze genes given the constraints of time in a lab session.


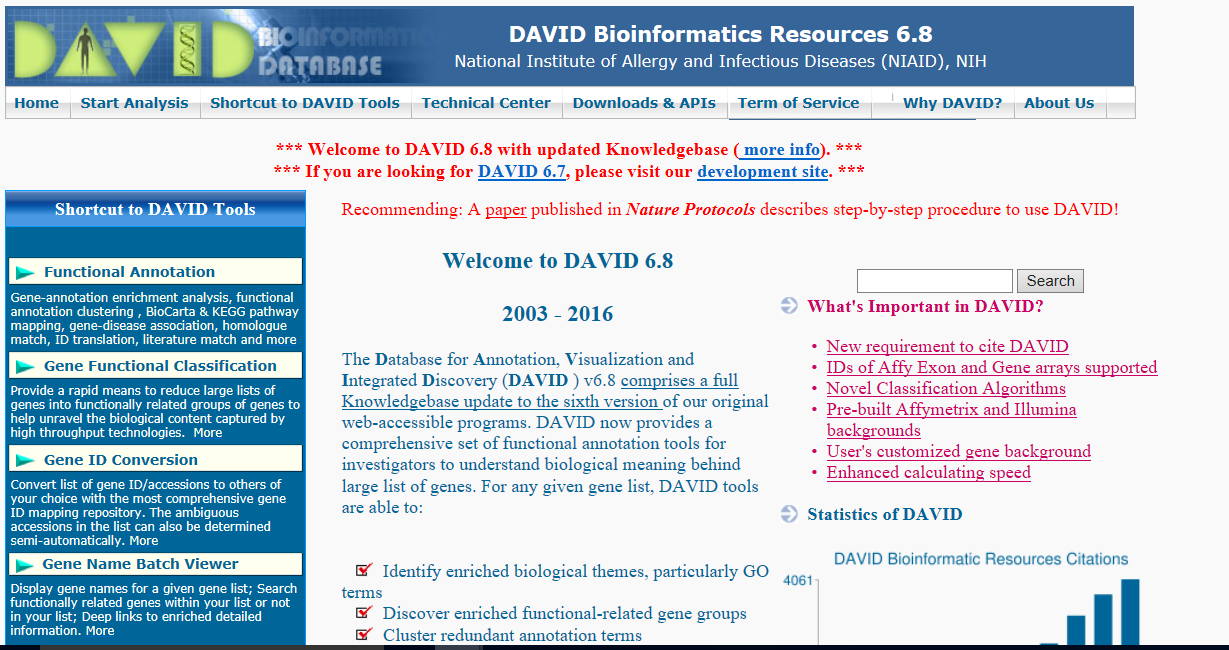


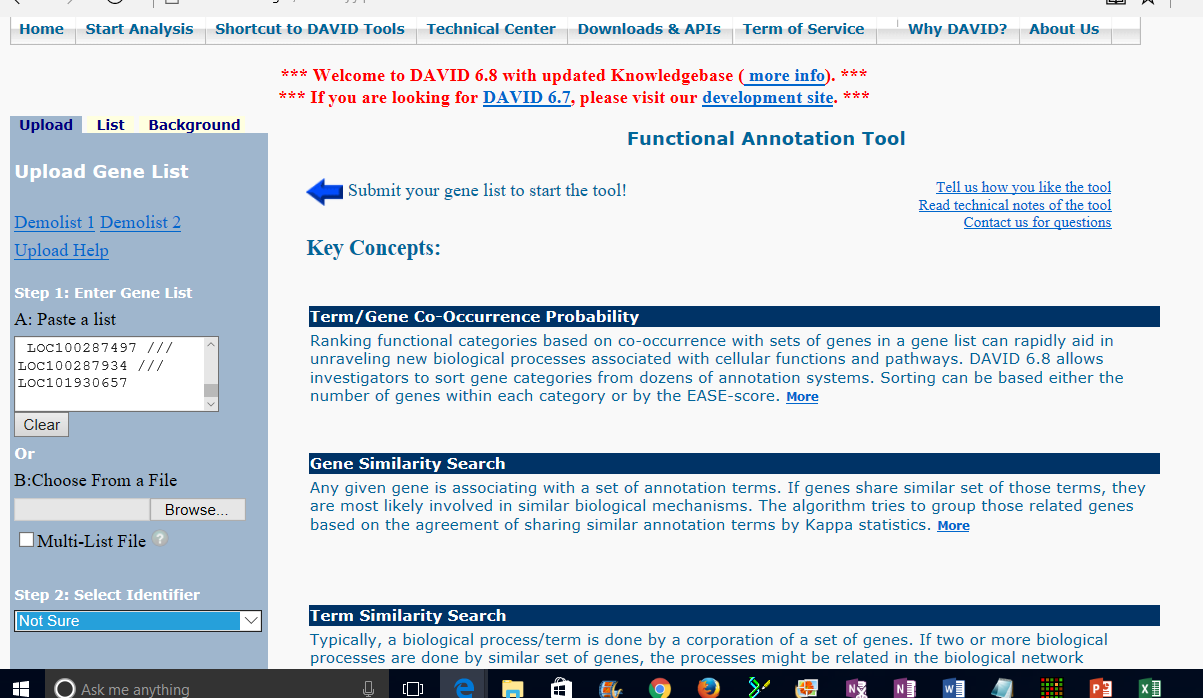


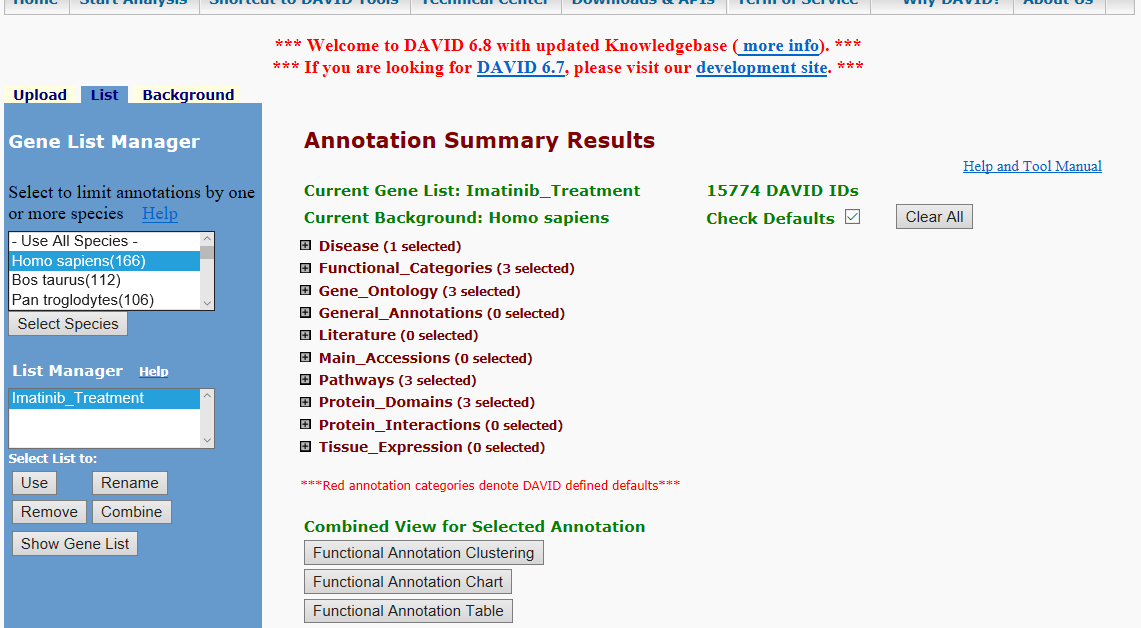


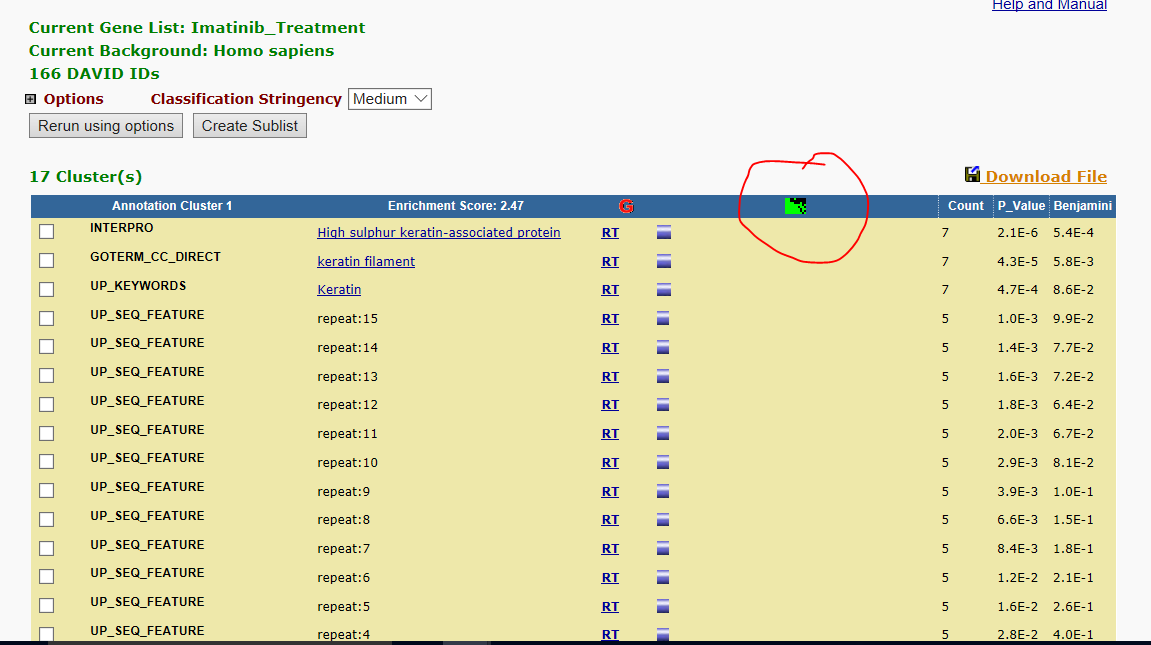


Answer:


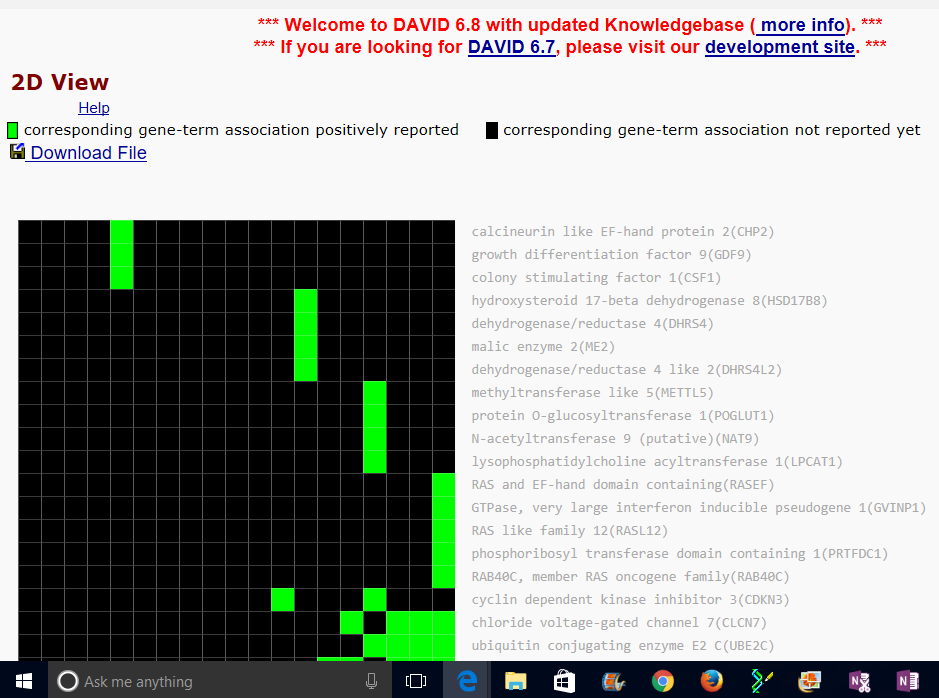

Supplement: S4 [file NIHMS1030899-supplement-S4.docx]
