# Supplementary material for: Investigating Cell Signaling with Gene Expression Datasets
Source: CourseSource. Author manuscript; Available in PMC 2020 Aug 26. (PMC7449260; doi:10.24918/cs.2019.1)
Supplement: S5 [file NIHMS1030899-supplement-S5.docx]

Supporting File S5: Example of a Worksheet for Guiding Group Discussions

Ask the students to watch videos through iBiology (total of 1 hour 30 minutes) and to identify answers to the questions below based on the video and the textbook. Small groups (3-5 students) are asked to prepare answers to specific questions to present to the class. The work is graded as participation credits.

<https://www.ibiology.org/biochemistry/protein-kinase/>

Dr. Susan Taylor, UC San Diego

Protein Kinases: Structure, Function, and Regulation

1. Illustrate the structure of a generic amino amino acid and identify the chemical group that distinguishes one amino acid from the other.
2. In brief, describe the chemical composition of amino acids.
3. Explain the classification of amino acids and give a least two examples for each group.
4. In proteins, which amino acids are subject to reversible phosphorylation?
5. What is the role or purpose of reversible protein phosphorylation?
6. What are protein kinases?
7. What are G protein-coupled receptors?
8. What is adenyl cyclase?
9. Define the term "human kinome". Is the human kinome different from, say, the mouse kinome?
10. What is glycogen? Briefly explain how the storage/utilization of glycogen is regulated.
11. Explain the role of protein kinase A in glycogen utilization.
12. Explain the mechanism of activation of a Ser/Thr kinase, e.g., PKA.
13. Identify the steps of signal transduction as they relate to glycogen hydrolysis, i.e., identify the reception, transduction and response stages.
14. Briefly explain the stages of a signal transduction pathway.
15. Discuss two distinct functions of ATP in cells and identify the phosphate or phosphates that is utilized in these functions.
16. What are the linkages between nucleotides in RNA and DNA called?
17. What are the linkages between amino acids in proteins called?
18. Explain the mechanism of activation of a Tyr kinase.
19. Discuss the role of conformational changes in the function of Src.
20. Why is Src described as a non-receptor tyrosine kinase?
