# Supplementary material for: Investigating Cell Signaling with Gene Expression Datasets
Source: CourseSource. Author manuscript; Available in PMC 2020 Aug 26. (PMC7449260; doi:10.24918/cs.2019.1)
Supplement: S6 [file NIHMS1030899-supplement-S6.docx]

**S6: Molecular Biology Laboratory Skills Survey**

1.The courses I have taken thus far in the biology curriculum sequence have emphasized research and research methodology.

A) I disagree

B) I somewhat agree

C I agree

D) I strongly agree

Indicate your level of familiarity with the following laboratory techniques and software packages.

2. Use of general cell and molecular biology equipment, such as micropipettes, balances,

pH meters and centrifuges.

A) I am not familiar with these pieces of equipment.

B) I know how to use at least one of these equipment.

C) I am somewhat familiar with at least two of these pieces of equipment.

D) I am proficient in using at least two of these equipment.

E) I am proficient in using all of the indicated equipment.

3. Regarding PubMed:

A) I have never used PubMed to search and retrieve information for my courses.

B) I have used PubMed for class work in at least one of my courses.

C) I am familiar with PubMed and I have used it in several classes.

D) I am familiar with PubMed and I use it regularly for class and/or research purposes.

4. Regarding BLAST:

A) I have never used BLAST to search and retrieve information for my courses.

'B) I have used BLAST for class work in at least one of my courses.

C) I am familiar with BLAST and I have used it in several classes.

D) I am familiar with BLAST and I use it regularly for class and/or research purposes.

E) I do regularly use the different algorithms of BLAST to search sequence databases.

5. Regarding protein structure analysis:

A) I have never used software and databases to analyze a protein structure.

B) I have analyzed at least one protein to determine structure-function relationship.

C) I am familiar with protein sequence, domain and structure databases.

D) I have used protein sequence, domain, and structure databases for class work and/or research.

E) I do regularly use protein sequence, domain, and structure databases for class work or research to search and retrieve information.

6. Regarding modeling:

A) I have never used software and databases to view protein structures

B) I have manipulated the structure of at least one protein using molecular graphics software

C) I am familiar with at least one molecular graphics software

D) I have used molecular graphics software for visualize and manipulate different protein structures.

E) I do regularly use molecular graphics software for research and I can help other

students

Select from the following to answer questions 7-20.

A) Unfamiliar with the term

B) I have used the technique in at least one laboratory session

C) I am fairly comfortable with the technique

D) I have used the technique on multiple occasions and I am able to interpret the data

E) I am proficient enough to assist other students with this technique

7. DNA or RNA isolation

8. Determination of concentration of macromolecules, such as proteins, RNA or DNA

9. Protein isolation

10. Protein analysis

11. PCR

12. SDS-PAGE

13. Plasmid isolation

14. Gene cloning

15. Cell culture

16. Immunoblotting

17. Spectrophotometry

18. Chromatography

19. Microscopy and other imaging techniques

20. Regarding data and data analysis

A) I am not familiar with statistical methods for testing hypotheses

B) I have used one statistical test to analyze data

C) I have used at least two statistical tests to analyze data

D) I can use multiple statistical tests to analyze data

E) I am proficient in statistical methods for data analysis and I can help other students.
